# Assortative Mating on Ancestry-Variant Traits in Admixed Latin American Populations

**DOI:** 10.3389/fgene.2019.00359

**Published:** 2019-04-24

**Authors:** Emily T. Norris, Lavanya Rishishwar, Lu Wang, Andrew B. Conley, Aroon T. Chande, Adam M. Dabrowski, Augusto Valderrama-Aguirre, I. King Jordan

**Affiliations:** ^1^School of Biological Sciences, Georgia Institute of Technology, Atlanta, GA, United States; ^2^IHRC-Georgia Tech Applied Bioinformatics Laboratory, Atlanta, GA, United States; ^3^PanAmerican Bioinformatics Institute, Cali, Colombia; ^4^Biomedical Research Institute, Cali, Colombia

**Keywords:** assortative mating, mate choice, genetic ancestry, admixture, population genomics, polygenic phenotypes

## Abstract

Assortative mating is a universal feature of human societies, and individuals from ethnically diverse populations are known to mate assortatively based on similarities in genetic ancestry. However, little is currently known regarding the exact phenotypic cues, or their underlying genetic architecture, which inform ancestry-based assortative mating. We developed a novel approach, using genome-wide analysis of ancestry-specific haplotypes, to evaluate ancestry-based assortative mating on traits whose expression varies among the three continental population groups – African, European, and Native American – that admixed to form modern Latin American populations. Application of this method to genome sequences sampled from Colombia, Mexico, Peru, and Puerto Rico revealed widespread ancestry-based assortative mating. We discovered a number of anthropometric traits (body mass, height, and facial development) and neurological attributes (educational attainment and schizophrenia) that serve as phenotypic cues for ancestry-based assortative mating. Major histocompatibility complex (MHC) loci show population-specific patterns of both assortative and disassortative mating in Latin America. Ancestry-based assortative mating in the populations analyzed here appears to be driven primarily by African ancestry. This study serves as an example of how population genomic analyses can yield novel insights into human behavior.

## Introduction

Mate choice is a fundamental dimension of human behavior with important implications for population genetic structure and evolution (Vandenburg, 1972; Buss, 1985; Robinson et al., 2017). It is widely known that humans choose to mate assortatively rather than randomly. That is to say that humans, for the most part, tend to choose mates that are more similar to themselves than can be expected by chance. Historically, assortative mating was based largely on geography, whereby partners were chosen from a limited set of physically proximal individuals (Cavalli-Sforza et al., 1994). Over millennia, assortative mating within groups of geographically confined individuals contributed to genetic divergence between groups, and the establishment of distincthuman populations, such as the major continental population groups recognized today (Rosenberg et al., 2002; Li et al., 2008; Genomes Project et al., 2015).

However, the process of geographic isolation followed by population divergence that characterized human evolution has not been strictly linear. Ongoing human migrations have continuously brought previously isolated populations into contact; when this occurs, the potential exists for once isolated populations to admix, thereby forming novel population groups (Hellenthal et al., 2014). Perhaps the most precipitous example of this process occurred in the Americas, starting just over 500 years ago with the arrival of Columbus in the New World (Mann, 2011). This major historical event quickly led to the co-localization of African, European and Native American populations that had been (mostly) physically isolated for tens of thousands of years (Jordan, 2016). As can be expected, the geographic reunification of these populations was accompanied, to some extent, by genetic admixture and the resulting formation of novel populations. This is particularly true for populations in Latin America, which often show high levels of three-way genetic admixture between continental population groups (Wang et al., 2008; Bryc et al., 2010; Ruiz-Linares et al., 2014; Montinaro et al., 2015; Rishishwar et al., 2015).

Nevertheless, modern admixed populations are still very much characterized by non-random assortative mating. Assortative mating in modern populations has been shown to rest on a variety of traits, including physical (stature and pigmentation) and neurological (cognition and personality) attributes. For example, numerous studies have demonstrated an influence of similarities in height and body mass on mate choice (Allison et al., 1996; Silventoinen et al., 2003; Robinson et al., 2017; Stulp et al., 2017). In addition, assortative mating has been observed for diverse neurological traits, such as educational attainment, introversion/extroversion and even neurotic tendencies (Merikangas, 1982; Mare, 1991; Hur, 2003; Salces et al., 2004; Domingue et al., 2014; Zou et al., 2015). Harder to classify traits related to personal achievement (income and occupational status) and culture (values and political leanings) also impact patterns of assortative mating (Merikangas, 1982; Kalmijn, 1994; Kandler et al., 2012). Odor is one of the more interesting traits implicated in mate choice, and it has been linked to so-called disassortative (or negative assortative) mating, whereby less similar mates are preferred. Odor-based disassortative mating has been attributed to differences in genes of the major histocompatibility (MHC) locus, which functions in the immune system, based on the idea that combinations of divergent human leukocyte antigen (HLA) alleles provide a selective advantage via elevated host resistance to pathogens (Wedekind et al., 1995; Chaix et al., 2008).

The traits that influence human mate choice are shaped by multiple factors with contributions from genes (G) and the environment (E) along with gene-by-environment (GxE) interactions. Studies that consider both genes and environment have shown different contributions of these factors to assortative mating patterns in human populations. Twin studies were initially used in an effort to tease apart the genetic and environmental contributions to mate choice (Lykken and Tellegen, 1993). Comparison of monozygotic and dizygotic twin pairs provided the first evidence for genetic influences on human mate choice, with 10–30% of the variance in mate choice explained by genetics compared to 10% shared environment and 60% unique environmental variance (Rushton and Bons, 2005). Subsequent twin design studies either did not find any strong genetic effects on patterns of assortative mating (Zietsch et al., 2011) or found genetic effects on assortative mating with very different relative contributions of genes versus environmental effects depending on the trait under consideration (Verweij et al., 2012). A more recent study leveraged genome-wide association study (GWAS) variants that influence height to show even more clear evidence for genetic effects on assortative mating (Li et al., 2017).

Ancestry is a particularly important determinant of assortative mating in modern admixed populations (Sebro et al., 2010; Zaitlen et al., 2017). Studies have shown that individuals in admixed Latin American populations tend to mate with partners that have similar ancestry profiles. For example, partners from both Mexican and Puerto Rican populations have significantly higher ancestry similarities than expected by chance (Risch et al., 2009; Zou et al., 2015). In addition, a number of traits that have been independently linked to assortative mating show ancestry-specific differences in their expression (Hancock et al., 2011). Accordingly, ancestry-based mate choice has recently been related to a limited number of physical (facial development) and immune-related (MHC loci) traits (Zou et al., 2015).

The studies that have uncovered the role of genetic ancestry in assortative mating among Latinos have relied on estimates of global ancestry fractions between mate pairs (Risch et al., 2009; Zou et al., 2015). Given the recent accumulation of numerous whole genome sequences from admixed Latin American populations – along with genome sequences from global reference populations (Genomes Project et al., 2015) – it is now possible to characterize local genetic ancestry for individuals from admixed American populations (Moreno-Estrada et al., 2013; Homburger et al., 2015; Rishishwar et al., 2015). In other words, the ancestral origins for specific chromosomal regions (haplotypes) can be assigned with high confidence for admixed individuals (Maples et al., 2013). For the first time here, we sought to evaluate the impact of local ancestry on assortative mating in admixed Latin American populations. Since the genetic variants that influence numerous phenotypes have been mapped to specific genomic regions, we reasoned that a focus on local ancestry could help to reveal the specific phenotypic drivers of ancestry-based assortative mating.

Our approach to this question entailed an integrated analysis of local genetic ancestry and the genetic architecture of a variety of human traits thought to be related to assortative mating. Assortative mating results in an excess of homozygosity, whereas disassortative mating yields excess heterozygosity. It follows that assortative (or disassortative) mating based on local ancestry would yield an excess (or deficit) of ancestry homozygosity at specific genetic loci. In other words, for a given population, a locus implicated in ancestry-based assortative mating would be more likely to have the same ancestry at both pairs of haploid chromosomes within individuals than expected by chance. We developed a test statistic – the assortative mating index (AMI) – that evaluates this prediction for individual gene loci, and we applied it to sets of genes that function together to encode polygenic phenotypes. We find evidence of substantial local ancestry-based assortative mating, and far less disassortative mating, for four admixed Latin American populations across a variety of anthropometric, neurological and immune-related phenotypes. Our approach also allowed us to assess the specific ancestry components that drive patterns of assortative and disassortative mating in these populations.

## Results

### Global and Local Genetic Ancestry in Latin America

**FIGURE 1:**
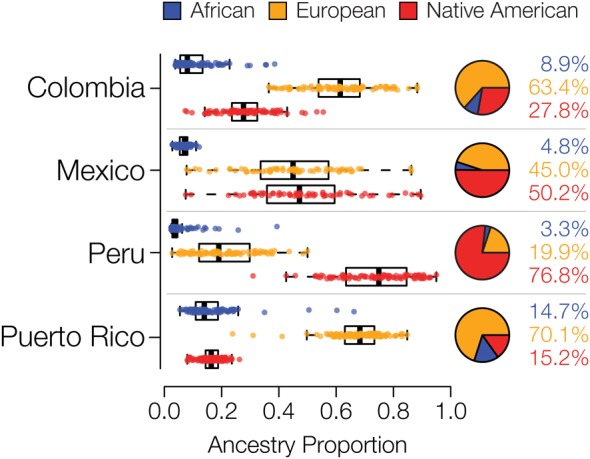
Genetic ancestry proportions for the admixed Latin American populations studied here. For each population, distributions and average values are shown for African (blue), European (orange), and Native American (red) ancestry.

We compared whole genome sequences from four admixed Latin American populations, characterized as part of the 1000 Genomes Project (1KGP) (Genomes Project et al., 2015) to genome sequences and whole genome genotypes from a panel of 34 global reference populations from Africa, Europe and the Americas (Table 1 and Supplementary Figure S1). The program ADMIXTURE (Alexander et al., 2009) was used to infer the continental genetic ancestry fractions – African, European and Native American – for individuals from the four Latin American populations (Supplementary Figure S2). Distributions of individuals’ continental ancestry fractions illustrate the distinct ancestry profiles of the four populations (Figure 1). Puerto Rico and Colombia show the highest European ancestry fractions along with the highest levels of three-way admixture. These two populations also have the highest African ancestry fractions, although all four populations have relatively small fractions of African ancestry. Peru and Mexico show more exclusively Native American and European admixture, with Peru having by far the largest Native American ancestry fraction.

**Table 1:** Human populations analyzed in this study.

	Dataset^a^	Geographical source	Short	*n*		Dataset^a^	Geographical source	*n*
	1KGP	Esan in Nigeria	ESN	99		HGDP	Pima in Mexico	14
			
	1KGP	Gambian in Western Division, The Gambia	GWD	113		HGDP	Maya in Mexico	21
			
	1KGP	Luhya in Webuye, Kenya	LWK	99		Reich et al.	Tepehuano in Mexico	25
			
Africa (*n* = 547)	1KGP	Mende in Sierra Leone	MSL	85		Reich et al.	Mixtec in Mexico	5
			
	1KGP	Yoruba in Ibadan, Nigeria	YRI	108		Reich et al.	Mixe in Mexico	17
			
	HGDP	Mandenka		22		Reich et al.	Zapotec in Mexico	43
			
	HGDP	Yoruba		21		Reich et al.	Kaqchikel in Guatemala	13
		
	1KGP	Finnish in Finland	FIN	99		Reich et al.	Kogi in Colombia	4
			
	1KGP	British in England and Scotland	GBR	90		Reich et al.	Waunana in Colombia	3
			
	1KGP	Iberian populations in Spain	IBS	107		Reich et al.	Embera in Colombia	5
			
Europe (*n* = 471)	1KGP	Toscani in Italy	TSI	107	Native American (*n* = 280)	Reich et al.	Guahibo in Colombia	6
			
	HGDP	Russian		25		Reich et al.	Piapoco in Colombia	7
			
	HGDP	Orcadian		15		Reich et al.	Inga in Colombia	9
			
	HGDP	French		28		Reich et al.	Wayuu in Colombia	11
		
	1KGP	Colombian in Medellin, Colombia	CLM	94		HGDP	Karitiana in Brazil	14
			
	1KGP	Peruvian in Lima, Peru	PEL	85		HGDP	Suruí in Brazil	8
			
Admixed (*n* = 347)	1KGP	Mexican Ancestry in LA, California	MXL	64		Reich et al.	Ticuna in Brazil	6
			
	1KGP	Puerto Rican in Puerto Rico	PUR	104		Reich et al.	Quechua in Peru	40
			
						Reich et al.	Aymara in Bolivia	23
						
						Reich et al.	Guarani in Paraguay	6

*Populations are organized into continental groups, for both ancestral and admixed Latin American populations, and the number of individuals in each population and group is shown. ^*a*^1KGP, 1000 Genomes Project; HGDP, Human Genome Diversity Panel; (Reich et al., 2012).*

The program RFMix (Maples et al., 2013) was used to infer local African, European and Native American genetic ancestry for individuals from the four admixed Latin American populations analyzed here. RFMix uses global reference populations to perform chromosome painting, whereby the ancestral origins of specific haplotypes are characterized across the entire genome for admixed individuals. Only haplotypes with high confidence ancestry assignments (≥99%) were taken for subsequent analysis. Examples of local ancestry assignment chromosome paintings for representative admixed individuals from each population are shown in Supplementary Figure S3. The overall continental ancestry fractions for admixed genomes calculated by global and local ancestry analysis are highly correlated, and in fact virtually identical, across all individuals analyzed here, in support of the reliability of these approaches to ancestry assignment (Supplementary Figure S4).

### Assortative Mating and Local Ancestry in Latin America

**FIGURE 2:**
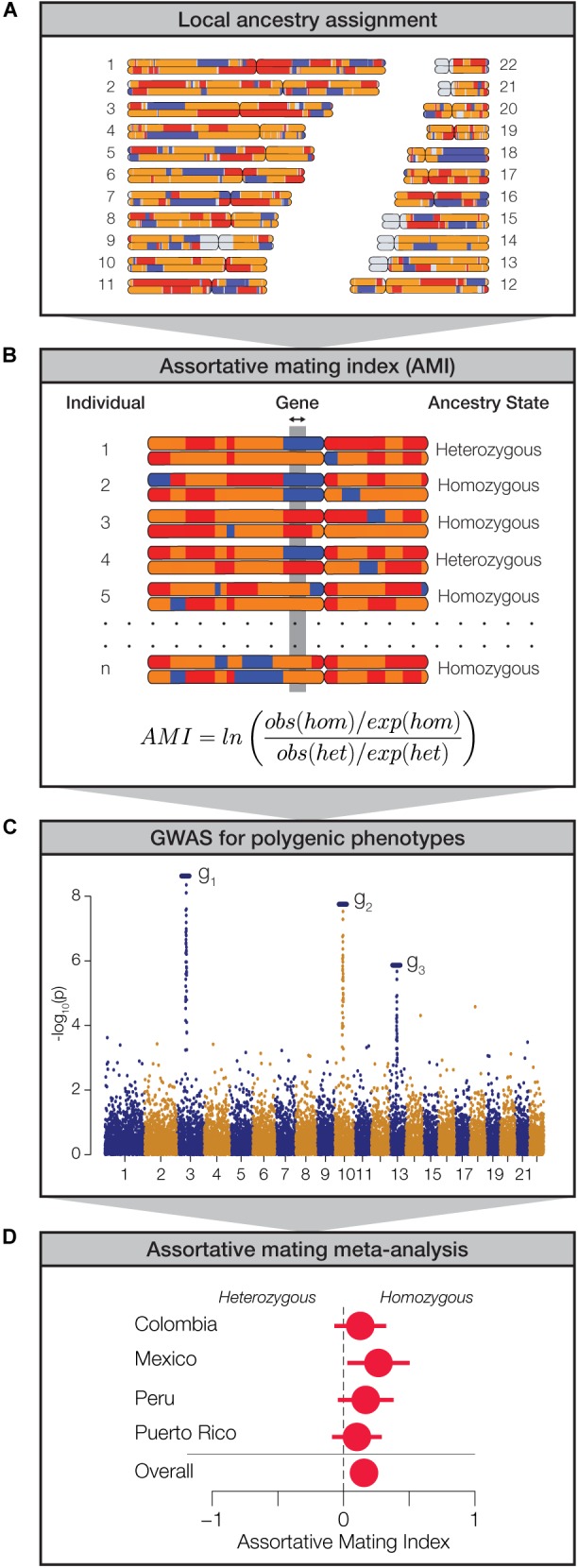
Approach used to measure assortative mating on local ancestry. **(A)** Local ancestry is assigned for specific haplotypes across the genome: African (blue), European (orange), and Native American (red). **(B)** Within individual genomes, genes are characterized as homozygous or heterozygous for local ancestry. For any given population, at each gene locus, the assortative mating index (AMI) is computed from the observed and expected counts of homozygous and heterozygous gene pairs. **(C)** Data from genome-wide association studies (GWAS) are used to evaluate polygenic phenotypes. **(D)** Meta-analysis of AMI values is used to evaluate the significance of ancestry-based assortative mating for polygenic phenotypes.

We analyzed genome-wide patterns of local ancestry assignment to assess the evidence for assortative mating based on local ancestry in Latin America (Figure 2A). For each individual, the ancestry assignments for pairs of haplotypes at any given gene were evaluated for homozygosity (i.e., the same ancestry on both haplotypes) or heterozygosity (i.e., different ancestry on both haplotypes) (Figure 2B). For each gene, across all four populations, the observed values of ancestry homozygosity and heterozygosity were compared to expected values in order to compute gene- and population-specific AMI values. AMI is computed as a log odds ratio as described in the Materials and Methods. The expected values of local ancestry homozygosity and heterozygosity used for the AMI calculations are based on a Hardy-Weinberg (HW) triallelic model with the three allele frequencies computed as the locus-specific ancestry fractions. High positive AMI values result from an excess of observed local ancestry homozygosity and are thereby taken to indicate assortative mating based on shared local genetic ancestry. Conversely, low negative AMI values indicate excess local ancestry heterozygosity and disassortative mating. We performed a series of controls to validate the performance of the AMI test statistic and the justification of the HW model for locus-specific ancestry. These controls are described in detail in the Supplementary Methods, and results of the controls can be seen in Supplementary Figures S5–S7.

While we were interested in exploring the relationship between local genetic ancestry and assortative mating, we recognized that mate choice is based on phenotypes rather than genotypes *per se*. Since phenotypes are typically encoded by multiple genes, expressed in the context of their environment, we used data from genome-wide association studies (GWAS) to identify sets of genes that function together to encode polygenic phenotypes (Figure 2C). We combined data from several GWAS database sources in order to curate a collection of 105 gene sets that have been linked to the polygenic genetic architecture of a variety of human traits. These gene sets range in size from 2 to 212 genes and include a total of 923 unique genes (Supplementary Figure S8). We focused on phenotypes that are known or expected to influence mate choice and thereby impact assortative mating patterns. These phenotypes fall into three broad categories: anthropometric traits (e.g., body shape, stature, and pigmentation), neurological traits (e.g., cognition, personality, and addiction), and immune response (HLA genes). Finally, we used a meta-analysis of the AMI values for the sets of genes that underlie each polygenic phenotype in order to evaluate the impact of local ancestry on assortative mating (Figure 2D).

**FIGURE 3:**
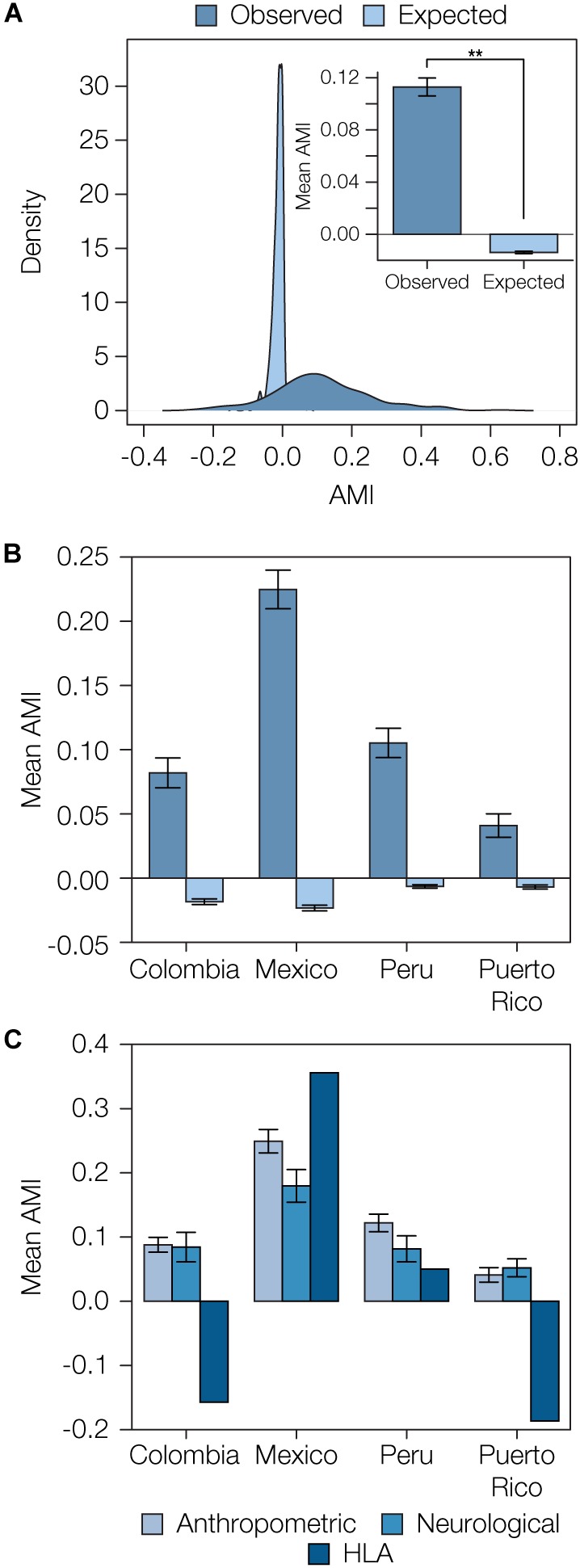
Overview of ancestry-based assortative mating in the four admixed Latin American populations analyzed here. **(A)** Distributions of observed and expected AMI values for all four populations. Inset: Mean observed and expected AMI values (±se) for all four populations. Significance between mean observed and expected AMI values (*P* = 8.12e-56) is indicated by two asterisks. **(B)** Observed and expected average AMI values (±se) across all polygenic phenotype gene sets are shown for each population. **(C)** Average AMI values (±se) for each population are shown for the three main phenotype functional categories characterized here: anthropometric, neurological, and human leukocyte antigen (HLA) genes.

We compared the distributions of observed AMI values versus those expected under random mating to assess the overall evidence for local ancestry-based assortative mating in Latin America. Expected AMI values were computed via permutation analysis by randomly combining pairs of haplotypes into 10,000 diploid individuals for each population to approximate random mating. The distribution of the expected AMI values under random mating is narrow and centered around 0, whereas the observed AMI values have a far broader distribution and tend to be positive (expected AMI μ = -0.01, σ = 0.03, observed AMI μ = 0.11, σ = 0.14; Figure 3A). When all four admixed Latin American populations are considered together, the mean observed AMI value is significantly greater than the expected mean AMI under random mating (*t* = 18.14, *P* = 8.12e-56). The same trend can be seen when all four populations are considered separately (Supplementary Figure S9). Mean observed AMI values vary substantially across populations, with Mexico showing the highest levels of local ancestry-based assortative mating and Puerto Rico showing the lowest (Figure 3B). There is also substantial variation seen for the extent of assortative mating among the three broad functional categories of phenotypes (Figure 3C). Local ancestry-based assortative mating is particularly variable for HLA genes, with high levels of assortative mating seen for Mexico and evidence for disassortative mating seen for Colombia and Puerto Rico. Anthropometric traits tend to show higher levels of local ancestry-based assortative mating across all four populations compared to neurological traits.

### Local Ancestry-Based Assortative Mating for Polygenic Phenotypes

When considered together, observed AMI levels are enriched for positive values compared to the expected values based on randomly paired haplotypes, indicative of an overall trend of assortative mating based on local ancestry in admixed Latin American populations (Figure 3A and Supplementary Figure S10). We evaluated polygenic phenotypes individually to look for the strongest examples of traits linked to local ancestry-based assortative mating and to evaluate traits that show either similar or variable assortative mating trends across populations. We computed AMI values for 105 polygenic phenotypes across the four populations; the expected and observed AMI values for all traits are shown in Supplementary Figure S10. As can be seen for the overall patterns of assortative mating, individual polygenic phenotypes show more extreme positive (for most cases) and negative (in a few cases) AMI values in the four admixed Latin American populations than can be expected for randomly mating populations.

However, as discussed previously, it is known that admixed Latin American populations mate assortatively based on genetic ancestry (Risch et al., 2009; Zou et al., 2015). This can be expected to lead to an overall excess of ancestry homozygosity genome-wide compared to expectations based on truly random mating, and we wanted to control for this as well when analyzing the assortative mating signals for specific traits. To do so, we performed an additional permutation analysis by choosing random sets of genes, of the same size as the observed trait-specific gene sets, and then computing AMI values and their significance levels for the randomly permuted gene sets. This allowed us to ask whether the polygenic phenotypes that have statistically significant AMI values show more extreme deviations than can be expected based on genome-wide signals of ancestry-based assortative mating. The results of this additional permutation control are described below in the context of the specific polygenic phenotypes that were found to have significant AMI values.

**FIGURE 4:**
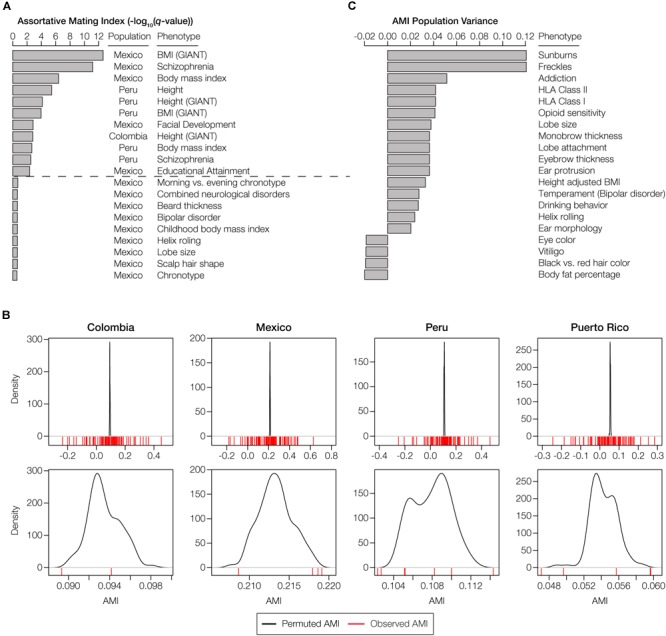
Phenotypes with statistically significant patterns of assortative mating within and among populations. **(A)** The top 20 phenotypes with the highest, and most statistically significant, assortative mating values (AMI) seen within any individual population. All AMI values shown are significant at *P* < 0.05, and the dashed line corresponds to a false discovery rate *q*-value cutoff of 0.05. **(B)** The observed AMI values for all trait-specific gene sets in each population (red lines) are compared to distributions of expected AMI values (black lines) based on random permutations of 10,000 gene sets. The top panels show the overall distributions of observed and permuted AMI values for each population, with steep peaks around the mean values for expected AMI. The bottom panels show a more narrow range of observed and expected AMI values for each population, which are centered around the population-specific mean expected AMI values. **(C)** The top 20 phenotypes with the highest or lowest, and most statistically significant, AMI variance levels across populations. Across population variance levels are normalized using the average AMI population variance level for all phenotypes. All AMI variance levels shown are significant at *q* < 0.05. The highest variance (most dissimilar patterns) of the AMI are at the top, while the lowest variance (most similar patterns) of AMI are at the bottom.

There are 11 polygenic phenotypes that yield statistically significant AMI values with the HW-based test, after correction for multiple tests, indicative of local ancestry-based assortative mating for specific traits (*q* < 0.05; Figure 4A). A number of other traits shown are marginally significant after correction for multiple testing. We compared the observed AMI values for the gene sets studied here to population-specific null distributions of expected AMI values generated using randomly permuted gene sets as described in the preceding paragraph (Figure 4B). This permutation test accounts for the genome-wide deviations from HW that occur due to ancestry-based assortative mating. The comparisons of the observed versus expected AMI values for this control indicate that the AMI signals that we observe here are trait-specific and cannot be attributed to the elevated levels of ancestry similarity seen for couples in admixed Latin American populations.

The majority of the statistically significant cases of assortative mating are seen in the Mexican and Peruvian populations (10 out of 11), and the anthropometric functional category is most commonly seen among the significant phenotypes (8 out of 11). Height and body mass index are the most commonly observed phenotypes among the significant cases, each appearing in two out of the four populations analyzed here (Colombia and Peru for height and Mexico and Peru for body mass index). The only neurological traits that show significant evidence of assortative mating are schizophrenia (Mexico and Peru) and educational attainment (Mexico). A number of other neurological and anthropometric traits in Mexico are marginally significant. Puerto Rico was the only population that did not show any individual phenotypes with significant evidence of assortative mating, consistent with its low overall AMI values (Figure 3B and Supplementary Figure S8). A list of these significant traits, including references to the literature where the trait single nucleotide polymorphism (SNP)-associations were originally reported, is provided in Supplementary Table S1. In addition to evaluating individual phenotypes for statistically significant AMI values, we also looked for polygenic phenotypes that showed the most similar or dissimilar patterns of assortative mating across the four admixed Latin American populations. The top 20 phenotypes with the highest and lowest population variance are shown in Figure 4C (all are statistically significant at *q* < 0.05). The polygenic phenotypes with the most variance in population-specific AMI values show more functional diversity compared to the phenotypes with the strongest signals for assortative mating. All three functional categories are represented among the highly population variant phenotypes, and the highly variant phenotypes consist of both assortative and disassortative mating cases (specifically the HLA genes that are described in more detail below). Neurological phenotypes are enriched among the variant cases, including temperament and several addiction-related phenotypes: opioid sensitivity and drinking behavior. A list of the population (in)variant traits, including references to the literature where the trait SNP-associations were originally reported, is provided in Supplementary Table S1.

**FIGURE 5:**
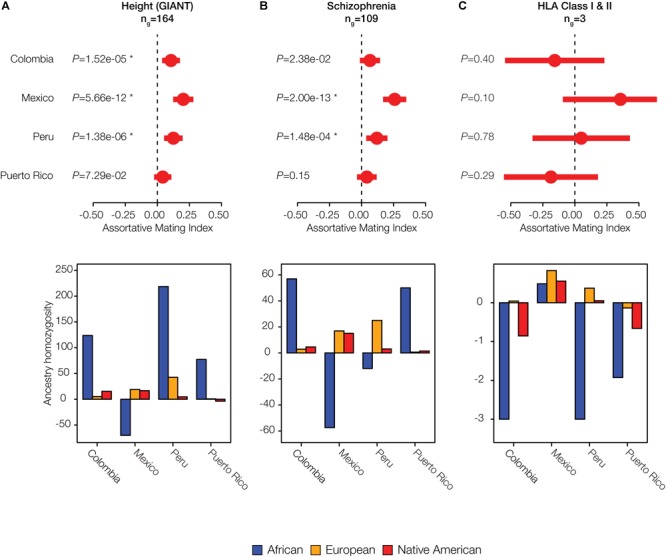
Individual examples of ancestry-based assortative and disassortative mating. Results of meta-analysis of (dis)assortative mating on polygenic phenotypes along with their ancestry drivers are shown for **(A)** an anthropometric trait: Height, **(B)** a neurological trait: Schizophrenia, and **(C)** the immune-related HLA class I and II genes. The meta-analysis plots show pooled AMI odds ratio values along with their 95% CIs and *P*-values. Stars indicate false discovery rate *q*-values < 0.05. The ancestry driver plots show the extent to which individual ancestry components – African (blue), European (orange), and Native American (red) – have an excess (>0) or a deficit (<0) of homozygosity.

Given the evidence of significant local ancestry-based assortative mating that we observed for a number of traits, we evaluated whether there were particular ancestry components that were most relevant to mate choice. In other words, we asked whether the excess counts of observed ancestry homozygosity or heterozygosity are linked to specific local ancestry assignments: African, European and/or Native American. For significant polygenic phenotype gene sets of interest, we computed the observed versus expected ancestry homozygosity for each ancestry separately across all genes in the set (Figure 5). Height is an anthropometric trait for which Colombia, Mexico, and Peru show significant evidence of assortative mating after correction for multiple tests (*q* < 0.05; Figure 5A), and Puerto Rico shows nominally significant assortative mating for this same trait (*P* < 0.05). In Colombia, Peru, and Puerto Rico, assortative mating for this polygenic phenotype is driven by an excess of African homozygosity, whereas in Mexico there is a lack of African homozygosity. The neurological disease schizophrenia shows statistically significant assortative mating in Mexico and Peru (*q* < 0.05), with marginally significant values in Colombia (Figure 5B). Patterns of assortative mating for this trait in Mexico and Peru are driven mainly by European ancestry, whereas Colombia and Puerto Rico show an excess of African ancestry homozygosity for this same trait.

Both Colombia and Puerto Rico show disassortative mating patterns for all HLA loci (both class I and II genes) (Figure 5C). The combined AMI values for the HLA loci are only marginally significant but they are among the lowest AMI values seen for any trait evaluated here (Supplementary Figure S10), and they are also highly variable among populations (Figure 4C). HLA loci in Colombia and Puerto Rico show a distinct lack of ancestry homozygosity for almost all ancestry components (Figure 5C). Mexico and Peru, on the other hand, have some evidence for assortative mating for the HLA loci; Mexico has the highest estimates of ancestry homozygosity at HLA loci for any of the four populations, and Peru has an excess of European and Native American ancestry homozygosity and a deficit of African homozygosity for these genes. Similar results for two additional anthropometric phenotypes are shown in Supplementary Figure S11: body mass index and facial development. These phenotypes show assortative mating in all four populations, with varying components of ancestral homozygosity driving the relationships. When these results are considered together, African ancestry consistently shows the strongest effect on driving assortative and disassortative mating in admixed Latin American populations (Figure 5 and Supplementary Figure S11).

**FIGURE 6:**
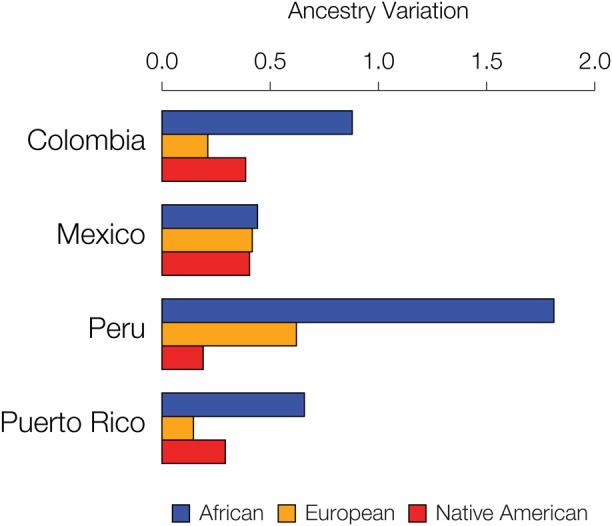
Inter-individual ancestry variance for the four admixed Latin American populations analyzed here. Variance among individuals for the African (blue), European (orange), and Native American (red) ancestry fractions within each population are shown.

We further evaluated the extent to which specific ancestry components may drive assortative mating patterns among admixed individuals by evaluating the variance of the three continental ancestry components among individuals within each Latin American population. Assortative mating is known to increase population variance for traits that are involved in mate choice; thus, the ancestry components that drive assortative mating in a given population are expected to show higher overall variance among individual genomes. African ancestry fractions show the highest variation among individuals for all four populations (Figure 6), consistent with the results seen for the five specific cases of assortative mating evaluated in Figure 5 and Supplementary Figure S11.

## Discussion

Assortative mating is a nearly universal human behavior, and scientists have long been fascinated by the subject (Vandenburg, 1972; Buss, 1985). Studies of assortative mating in humans have most often entailed direct measurements of traits – such as physical stature, education, and ethnicity – followed by correlation of trait values between partners. Decades of such studies have revealed numerous, widely varying traits that are implicated in mate choice and assortative mating. Studies of this kind typically make no assumptions regarding, nor have any knowledge of, the genetic heredity of the traits under consideration. Moreover, the extent to which the expression of these traits varies among human population groups has largely been ignored.

The first attempts to evaluate the genetic contributions to assortative mating entailed twin studies, whereby the similarity of mate choice for dizygotic versus monozygotic twins were compared (Lykken and Tellegen, 1993). While twin studies did uncover a genetic contribution to variance in human mate choice, they often yielded widely inconsistent results. This was true for both the overall extent of heritability in mate choice, which ranged from 0 to 30% (Rushton and Bons, 2005; Zietsch et al., 2011), and the relative amounts of genetic versus environmental contributions across the different traits implicated in mate choice (Verweij et al., 2012).

More recent studies of assortative mating, powered by advances in human genomics, have begun to explore the genetic architecture underlying the human traits that form the basis of mate choice in more detail (Domingue et al., 2014; Robinson et al., 2017). In addition, recent genomic analyses have underscored the extent to which human genetic ancestry influences assortative mating (Risch et al., 2009; Zou et al., 2015; Zaitlen et al., 2017). However, until this time, these two strands of inquiry have not been brought together. The approach that we developed for this study allowed us to directly assess the connection between local genetic ancestry – i.e., ancestry assignments for specific genome regions or haplotypes – and the human traits that serve as cues for assortative mating.

Previous studies on human mate choice have demonstrated pronounced sex differences in mate preference; for example, females value earning capacity more in potential mates, whereas males value reproductive capacity, as inferred from youth and physical attractiveness (Buss, 1989; Geary, 2000). It should be noted that the genome-based approach that we employed here does not allow us to consider sex differences in mate preference since we are essentially observing the effects of assortative mating on the offspring of mate pairs, by comparing ancestry homozygosity levels in the genomes of all individuals, rather than directly observing mate choice in couples.

Our approach relies on the well-established principle that assortative mating results in an excess of genetic homozygosity (Sebro et al., 2010). However, we do not analyze homozygosity of specific genetic variants *per se*, as is normally done; rather we evaluate excess homozygosity, or the lack thereof, for ancestry-specific haplotypes (Figure 2B). By merging this approach with data on the genetic architecture of polygenic human phenotypes, we were able to uncover specific traits that inform ancestry-based assortative mating. This is because, when individuals exercise mate choice decisions based on ancestry, they must do so using phenotypic cues that are ancestry-associated. In other words, ancestry-based assortative mating is, by definition, predicated upon traits that vary in expression among human population groups (Supplementary Figure S12). An obvious example of this is skin color (Hancock et al., 2011), and studies have indeed shown skin color to be an important feature of assortative mating (Frisancho et al., 1981; Malina et al., 1983; Trachtenberg et al., 1985; Procidano and Rogler, 1989). It follows that the assortative mating traits that our study uncovered in admixed Latin American populations must be both genetically heritable and variable among African, European and Native American population groups.

The traits we found to influence ancestry-based assortative mating vary among the continental population groups that admixed to form modern Latin American populations (Supplementary Table S2). For example, the anthropometric traits found in our study – body mass, height and facial development – are both heritable and known to vary among ancestry groups. This implies that the genetic variants that influence these traits should also vary among these populations. Accordingly, it is readily apparent that mate choice decisions based on these physical features could track local genetic ancestry. Interpretation of the neurological traits that show evidence of local ancestry-based assortative mating – schizophrenia and educational attainment – is not quite as straightforward. For schizophrenia, it is far more likely that we are analyzing genetic loci associated with a spectrum of personality traits that influence assortative mating, as opposed to mate choice based on full-blown schizophrenia, and indeed personality traits are widely known to impact mate choice decisions (Merikangas, 1982; Hur, 2003; Kandler et al., 2012). In addition, since schizophrenia prevalence does not vary greatly world-wide (Saha et al., 2005), it is more likely that ancestry-based assortative mating for this trait is tracking an underlying endophenotype rather than the disease itself. While educational attainment outcomes are largely environmentally determined, recent large-scale GWAS studies have uncovered a substantial genetic component to this trait, which is distributed among scores of loci across the genome (Rietveld et al., 2013, 2014; Davies et al., 2016; Okbay et al., 2016). The population distribution of education-associated variants is currently unknown, but our results suggest the possibility of ancestry-variation for some of them. Indeed, the average allele frequencies for the variants that influence our top four traits of interest – height, BMI, schizophrenia, and educational attainment – show significant variation among ancestry groups (Supplementary Figure S12).

Mate choice based on divergent MHC loci, apparently driven by body odor preferences, is the best known example of human disassortative mating (Wedekind et al., 1995). However, studies of this phenomenon have largely relied on ethnically homogenous cohorts. In one case where females were asked to select preferred MHC-mediated odors from males of a different ethnic group, they actually preferred odors of males with more similar MHC alleles (Jacob et al., 2002). Another study showed differences in MHC-dependent mate choice for human populations with distinct ancestry profiles (Chaix et al., 2008). Ours is the first study that addresses the role of ancestry in MHC-dependent mate choice in ethnically diverse admixed populations. Unexpectedly, we found very different results for MHC-dependent mate choice among the four Latin American populations that we studied. In fact, AMI values for the HLA loci are among the most population variable for any trait analyzed here (Figure 4C). Mexico and Peru show evidence of assortative mating at HLA loci, whereas Colombia and Puerto Rico show evidence for disassortative mating (Figure 5C). Interestingly, disassortative mating for HLA loci in Colombia and Puerto Rico is largely driven by African ancestry, and these two populations have substantially higher levels of African ancestry compared to Mexico and Peru. The population- and ancestry-specific dynamics of MHC-dependent mate choice revealed here underscore the complexity of this issue. Given the complexity of the results reported here, particularly as they relate to differences among populations, it should also be noted that anomalous patterns of linkage disequilibrium at MHC loci could confound the analysis at this region.

Assortative mating alone is not expected to change the frequencies of alleles, or ancestry fractions in the case of our study, within a population. Assortative mating does, however, change genotype frequencies, resulting in an excess of homozygous genotypes. Accordingly, ancestry-based assortative mating is expected to yield an excess of homozygosity for local ancestry assignments (i.e., ancestry-specific haplotypes) (Figure 2B). By increasing homozygosity in this way, assortative mating also increases the population genetic variance for the traits that influence mate choice. In other words, assortative mating will lead to more extreme, and less intermediate, phenotypes than expected by chance. This population genetic consequence of assortative mating allowed us to evaluate the extent to which specific continental ancestries drive mate choice decisions in admixed populations, since specific ancestry drivers of assortative mating are expected to have increased variance. We found that the fractions of African ancestry have the highest variance among individuals for all four populations, consistent with the idea that traits that are associated with African ancestry drive most of the local ancestry-based assortative mating seen in this study (Figure 6).

It is important to reiterate that previous studies have shown evidence for assortative mating on both genetic ancestry and specific traits; accordingly, ancestry-based population stratification could lead to the appearance of trait-based assortative mating (Sebro et al., 2017). For example, assortative mating occurs among European-Americans along a North-South European ancestry cline, which happens to mirror the cline in height along this same axis (Sebro et al., 2010). This begs the question as to whether similarities in height among European-American couples is due to ancestry or due to the trait itself. Studies have shown conflicting results regarding this question. On the one hand, genetic ancestry (population stratification) alone was posited to account for observed patterns of assortative mating in the US (Abdellaoui et al., 2014), whereas assortative mating for height was observed within distinct US population groups, independent of their ancestry (Li et al., 2017). Here, we have tried to tease apart the overall effects of ancestry-based assortative mating versus trait-specific mate choice by permuting random sets of genes and re-computing our AMI test statistic for each of the four Latin American populations analyzed here. This procedure allowed us to control for the background levels of local ancestry homozygosity in these populations, which could have been generated by ancestry-based assortative mating alone. We used this control to parameterize the significance levels for the test statistic that we used to discover trait-specific ancestry-based assortative mating (Figure 4B). In other words, we only find a specific trait to be implicated in ancestry-based assortative mating if the levels of ancestry homozygosity for the genes associated with that trait are significantly higher than the genome-wide background levels. In this sense, we have shown how these specific traits may serve as cues that underlie, to some extent, ancestry influenced mate choice in Latin American populations. A corollary to this conclusion is the fact that ancestry- and trait-based assortative mating cannot be completely disentangled for modern admixed populations. Rather, the variance in the expression of these traits across ancestry groups may in fact be an informative marker for individuals’ ancestral origins.

The confluence of African, European and Native American populations that marked the conquest and colonization of the New World yielded modern Latin American populations that are characterized by three-way genetic admixture (Wang et al., 2008; Bryc et al., 2010; Ruiz-Linares et al., 2014; Montinaro et al., 2015; Rishishwar et al., 2015). Nevertheless, mate choice in Latin America is far from random (Risch et al., 2009; Zou et al., 2015). Indeed, our results underscore the prevalence of ancestry-based assortative mating in modern Latin American societies. The local ancestry approach that we developed provided new insight into this process by allowing us to hone in on the phenotypic cues that underlie ancestry-based assortative mating. Our method also illuminates the specific ancestry components that drive assortative mating for different traits and makes predictions regarding traits that should vary among continental population groups.

## Materials and Methods

### Whole Genome Sequences and Genotypes

Whole genome sequence data for the four admixed Latin American populations studied here were taken from the Phase 3 data release of the 1000 Genomes Project (1KGP) (Genomes Project et al., 2015). Whole genome sequence data and genotypes for the putative ancestral populations (Africa, Europe, and the Americas) were taken from the 1KGP, the Human Genome Diversity Project (Li et al., 2008) (HGDP), and a previous study on Native American genetic ancestry (Reich et al., 2012).

Whole genome sequence data and genotypes were merged, sites common to all datasets were kept, and SNP strand orientation was corrected as needed, using PLINK version 1.9 (Chang et al., 2015). The resulting dataset consisted of 1,645 individuals from 38 populations with variants characterized for 239,989 SNPs. The set of merged SNP genotypes was phased, using the program SHAPEIT version 2.r837 (Delaneau et al., 2013), with the 1KGP haplotype reference panel. This phased set of SNP genotypes was used for local ancestry analysis. PLINK was used to further prune the phased SNPs for linkage, yielding a pruned dataset containing 58,898 linkage-independent SNPs. This pruned set of SNP genotypes was used for global ancestry analysis.

### Global and Local Ancestry Analysis

To infer continental (global) ancestry of the four admixed Latin American populations, ADMIXTURE (Alexander et al., 2009) was run on the pruned SNP genotype dataset (*n* = 58,898). ADMIXTURE was run using *K* = 4, yielding African, European, Asian and Native American ancestry fractions of each admixed individual; the final Asian and Native American fractions were summed to determine the Native American fraction of each individual. For local ancestry analysis of the admixed Latin American populations, the program RFMix (Maples et al., 2013) version 1.5.4 was run in the PopPhased mode with a minimum node size of 5 and the “usereference-panels-in-EM” option with 2 EM iterations for each individual in the dataset using the phased SNP genotypes (*n* = 239,989). Continental African, European, and Native American populations were used as reference populations, and contiguous regions with the same ancestry assignment, i.e., ancestry-specific haplotypes, were delineated where the RFMix ancestry assignment certainty was at least 99%. The timing of admixture events was analyzed using the program TRACTS (Gravel, 2012; Gravel et al., 2013) with the local ancestry haplotype assignments from RFMix. TRACTS was used to evaluate possible admixture timings across 1,000 bootstrap attempts, with the most likely series of admixture events chosen to represent each population.

Autosomal NCBI RefSeq coding genes were accessed from the UCSC Genome Browser and mapped to the ancestry-specific haplotypes characterized for each admixed Latin American individual. For each diploid genome analyzed here, individual genes can have 0, 1, or 2 ancestry assignments depending on the number of high confidence ancestry-specific haplotypes at that locus. Our assortative mating index (AMI, see below) can only be computed for genes that have 2 ancestry assignments in any given individual, i.e., cases where the ancestry is assigned for both copies of the gene. Thus, for each Latin American population p, the mean ($\bar{x_{p}}$), and standard deviation (sd_p_) of the number of genes with 2 ancestry assignments were calculated and used to compute an ancestry genotype threshold for the inclusion of genes in subsequent analyses. Genes were used in subsequent assortative mating analyses only if they were present above the ancestry genotype threshold of $\bar{x_{p}}$ - sd_p_.

### Gene Sets for Polygenic Phenotypes

The polygenic genetic architectures of phenotypes that could be affected by assortative mating were characterized using a variety of studies taken from the NHGRI-EBI GWAS Catalog (MacArthur et al., 2017), the Genetic Investigation of ANthropometric Traits (GIANT) consortium^1^, and PubMed literature sources.

For each polygenic phenotype, all SNPs previously implicated at genome-wide significance levels of *P* ≤ 5 × 10^-8^ were collected as the phenotype SNP set. The gene sets for the polygenic phenotypes were collected by directly mapping trait-associated SNPs to genes. SNPs were used to create a gene set only if the SNP fell directly within a gene and thus no intergenic SNPs were used in creating gene sets. Gene sets from the GWAS Catalog were mapped from SNPs using EBI’s in-house pipeline. Sets from GIANT were mapped according to specifications of each individual paper. Gene sets from literature searching were mapped using NCBI’s dbSNP. For each Latin American population, phenotype gene sets were filtered to only include genes that passed the ancestry genotype threshold, as described previously. Finally, the polygenic phenotype gene sets were filtered based on size, so that all polygenic phenotypes included two or more genes.

Linkage disequilibrium (LD) pruning was performed using the program PLINK to ensure that gene sets consisted of independent genes. LD pruning was done using pairwise *r*^2^-values between genic SNPs for all pairs of genes in any given set. For any pair of genes with SNP *r*^2^ > 0.1, only one member of the pair was retained for further analysis. The final data set contains gene sets for 105 polygenic phenotypes, hierarchically organized into three functional categories, including 923 unique genes (haplotypes) (Supplementary Figure S7).

### Assortative Mating Index (AMI)

To assess local ancestry-based assortative mating, we developed the AMI, a log odds ratio test statistic that computes the relative local ancestry homozygosity compared to heterozygosity for any given gene. Ancestry homozygosity occurs when both genes in a genome have the same local ancestry, whereas ancestry heterozygosity refers to a pair of genes in a genome with different local ancestry assignments. The AMI is calculated as:

$$\begin{array}{c} AMI=ln\left(\frac{obs\left(hom\right)/exp\left(hom\right)}{obs\left(het\right)/exp\left(het\right)}\right) \end{array}$$

where *obs(hom)/exp(hom)* is the ratio of the observed and expected local ancestry homozygous gene pairs and *obs(het)/exp(het)* is the ratio of the observed and expected local ancestry heterozygous gene pairs.

The observed values of local ancestry homozygous and heterozygous gene pairs are taken from the gene-to-ancestry mapping data for each gene in each population. The expected values of local ancestry homozygous and heterozygous gene pairs are calculated for each gene in a population using a triallelic Hardy-Weinberg (HW) model, in which the gene-specific local ancestry assignment fractions are taken as the three allele frequencies. For the African (a), European (e), and Native American (n) gene-specific local ancestry assignment fractions in a population, the HW expected genotype frequencies are: (a + e + n)^2^ or a^2^ + 2ae + e^2^ + 2an + 2en + n^2^. Accordingly, the expected frequency of homozygous pairs is a^2^ + e^2^ + n^2^ and the expected frequency of heterozygous pairs is 2ae + 2an + 2en. For each gene, in each population, the expected homozygous and heterozygous frequencies are multiplied by the number of individuals with two ancestry assignments for that gene to yield the expected counts of gene pairs in each class.

For each polygenic phenotype, a meta-analysis of gene-specific AMI values was conducted to evaluate the effect of all of the genes involved in the phenotype on assortative mating, using the metafor (Viechtbauer, 2010) package in R. 95% confidence intervals for each gene, meta-gene AMI values, significance *P*-values, and false discovery rate *q*-values, were computed using the Mantel-Haenszel method under a fixed-effects model.

### Controls for Evaluating the Assortative Mating Index (AMI)

Four different controls were used to evaluate the design and performance of the AMI test statistic: (1) a control for the use of HW as a null model in the AMI test statistic, (2) a permutation analysis to evaluate expected AMI values under random mating, (3) a population genetic simulation to evaluate the power of the AMI test statistic to detect ancestry-based assortative mating and its dependence on the different ancestry combinations of the populations we analyzed, and (4) a permutation of random gene sets to generate null distributions of AMI values expected given the observed genome-wide signals of ancestry-based assortative mating. Each of these control analyses is described in detail in the Supplementary Materials and Methods.

### Ancestry-Specific Drivers of Assortative Mating

For each significant polygenic phenotype of interest, we identified the ancestry component related to mate choice by calculating the ancestry homozygosity ($AH_{\mathrm{phenotype}}^{\mathrm{anc}}$) for all genes for each ancestry at the given phenotype. The ancestry homozygosity was calculated as:

$$\begin{array}{c} AH_{\mathrm{phenotype}}^{\mathrm{anc}}=\;\sum_{g\;\in \;\mathrm{genes}\;\mathrm{in}\;\mathrm{the}\;\mathrm{phenotype}}\left(\frac{obs_{g}^{\mathrm{anc}}-\;exp_{g}^{\mathrm{anc}}}{exp_{g}^{\mathrm{anc}}}\right) \end{array}$$

where anc is one of the three ancestries – African, European or Native American, g ∈ genes in the phenotype are all of the genes involved in the polygenic phenotype, $obs_{g}^{\mathrm{anc}}$ is the number of observed homozygous genes for gene g coming from anc, and $exp_{g}^{\mathrm{anc}}$ is the number of expected homozygous genes for gene g coming from anc (as calculated using a triallelic Hardy–Weinberg model).

### Statistical Significance Testing

Significance testing for the difference between the observed and expected AMI distributions (for both random mating and assortative mating) was completed using the t-test package in R. The metafor package, used for calculating the meta-analysis AMI values, also calculates a *P*-value and a false discovery rate *q*-value to correct for multiple statistical tests, which were used for identifying polygenic phenotypes that are significantly influenced by local ancestry-based assortative mating in each Latin American population. The variance of AMI values across the four populations for each phenotype was calculated as it is implemented in R and used for identifying phenotypes that had highly similar (minimal variance) or highly dissimilar (maximal variance) local ancestry-based assortative mating patterns. The coefficient of variation was used to measure the inter-individual variance for each of the three continental ancestry components within the four admixed Latin American populations analyzed here.

## Ethics Statement

All data were sourced from publicly-available databases and do not require additional ethics approval.

As per the Data Availability section of the manuscript, data can be access from: 1000 Genomes Project data are available from http://www.internationalgenome.org/data/

Human Genome Diversity Project data are available from http://www.hagsc.org/hgdp/

Previously published Native American genotype data can be accessed from a data use agreement governed by the University of Antioquia as previously described (Reich et al., 2012).

## Author Contributions

EN conducted all of the ancestry-based assortative mating analyses. LR performed the permutation and simulation analyses. LW participated in the assortative mating analysis for individual phenotypes. ABC performed the genetic ancestry analyses. EN, ATC, AD, and AV-A curated the GWAS SNP associations and polygenic phenotype gene sets. EN, LR, LW, and ABC generated the manuscript figures. IJ conceived of, designed and supervised the project. EN, LR, and IJ wrote the manuscript. All authors read and approved the final manuscript.

## Conflict of Interest Statement

The authors declare that the research was conducted in the absence of any commercial or financial relationships that could be construed as a potential conflict of interest.

## Footnotes

1 http://portals.broadinstitute.org/collaboration/giant/index.php/GIANT_consortium
