# Supplementary material for: Assortative Mating on Ancestry-Variant Traits in Admixed Latin American Populations
Source: Front Genet. 2019 Apr 24;10:359. doi: 10.3389/fgene.2019.00359 (PMC6491930; doi:10.3389/fgene.2019.00359)
Supplement: Supplementary file 1 [file Data_Sheet_1.PDF]

Supplementary Material

Assortative mating on ancestry-variant traits in admixed Latin American populations

Emily T. Norris, Lavanya Rishishwar, Lu Wang, Andrew B. Conley, Aroon T. Chande, Adam M. Dabrowski, Augusto Valderrama-Aguirre, I. King Jordan

1. Supplementary Materials and Methods 3
  Controls for evaluating the assortative mating index (AMI) 3
    Control 1: Evaluation of Hardy-Weinberg (HW) 3
    Control 2: Permutation of random mating 3
    Control 3: Population genetic simulation of assortative mating 3
    Control 4: Permutation test for ancestry-based assortative mating 5
2. Supplementary Results 6
  Figure S1. Global locations of the populations analyzed in this study. 6
  Figure S2. Three-way continental genetic ancestry for the four admixed Latin American populations analyzed in this study. 7
  Figure S3. Local ancestry assignment with chromosome painting. 8
  Figure S4. Comparison of ancestry fractions estimated by ADMIXTURE (global ancestry) versus RFMix (local ancestry). 9
  Control 1: Evaluation of Hardy-Weinberg (HW) 10
  Control 2: Permutation of random mating 10
  Control 3: Population genetic simulation of assortative mating 10
  Control 4: Permutation test for ancestry-based assortative mating 11
  Figure S5. Genome-wide patterns of homozygosity and heterozygosity for the four admixed Latin American populations analyzed in this study. 12
  Figure S6. Admixture timing for the four admixed Latin American populations analyzed in this study. 13
  Figure S7. Simulation of the assortative mating index (AMI) test statistic under assortative mating. 14
  Figure S8. Polygenic phenotypes taken from genome-wide association studies (GWAS). 15
  Figure S9. Distributions of observed (dark blue) versus expected (light blue) AMI values for the four admixed Latin American populations analyzed here. 16

Figure S10. Assortative mating index (AMI) values for all phenotypes across all four populations analyzed here. 17

Figure S11. Individual examples of ancestry-based assortative mating. 18

Figure S12. Genetic variation in trait-specific SNP frequencies across continental ancestry groups. 19

Table S1. References and values for phenotypes with significant AMI values and population variance. 20

Table S2. Ancestry differences for phenotypes implicated in assortative mating (i.e. mate choice) in admixed Latin American populations. 24

3. References 25

1. Supplementary Materials and Methods

Controls for evaluating the assortative mating index (AMI)

Four different controls were used to evaluate the design and performance of the AMI test statistic: (1) a control for the use of HW as a null model in the AMI test statistic, (2) a permutation analysis to evaluate expected AMI values under random mating, (3) a population genetic simulation to evaluate the power of the AMI test statistic to detect ancestry-based assortative mating and its dependence on the different ancestry combinations of the populations we analyzed, and (4) a permutation of random gene sets to generate null distributions of AMI values expected given the observed genome-wide signals of ancestry-based assortative mating. Each of these control analyses is described in the following text.

Control 1: Evaluation of Hardy-Weinberg (HW)

HW was intended as a null model against which to test our observed data; nevertheless, it is possible that local ancestry will deviate from HW depending on population history and demography. To control for this possibility, we tested for evidence of (1) the Wahlund effect, which is expected to yield an excess of homozygosity, and (2) recent admixture, which is expected to yield an excess of heterozygosity. The Wahlund effect, along with the genome-wide distributions of homozygosity and heterozygosity, were measured using the genome-wide distribution of the parameter Φ, where $\Phi = 2pq/\sqrt{p^2 \times q^2}$ with p and q representing ancestry fractions (Lachance, 2008). Genome-wide distributions of heterozygosity were evaluated to look for recent admixture yielding very high heterozygosity. In addition, the admixture timing for the four populations was analyzed using the distributions of the ancestry-specific haplotype lengths with the program TRACTS as described in the Global and local ancestry analysis subsection of the Materials and Methods.

Control 2: Permutation of random mating

A standard permutation testing framework was adopted for the approximation of random mating in each of the four Latin American populations. Random mating was approximated by randomly combining pairs of individual phased haplotypes from a population to yield permuted diploid genotypes. Haploid chromosomes were permuted randomly within each population using the Fisher-Yates shuffle. After permutation of the chromosomes, per gene AMI values were re-calculated for all genes passing the population-specific ancestry genotyping thresholds. The permutations were completed 20 times, and the population-specific mean AMI values for each gene were taken as the permuted AMI for the gene. This mean permuted AMI per gene was used in AMI meta-analysis for each gene set to determine expected AMI values.

Control 3: Population genetic simulation of assortative mating

To validate the performance of the AMI test statistic, we adopted a population genetic model that simulates assortative mating in the four Latin American populations under Hardy-Weinberg

equilibrium, with a fraction of the population mating assortatively. For each gene in a given population, the present-day local ancestry assignment fractions are used as the starting ancestral proportions: African = a, European = e, Native American = n. Using a triallelic Hardy-Weinberg model, taking the ancestral proportions as the allele frequencies, the ancestry genotype frequencies for a given gene at the starting generation are calculated as:

$$P_{aa} = a^2$$

$$P_{ae} = 2ae$$

$$P_{an} = 2an$$

$$P_{ee} = e^2$$

$$P_{en} = 2en$$

$$P_{nn} = n^2$$

where P_{aa} = African-African genotype, P_{ae} = African-European genotype, P_{an} = African-Native American genotype, P_{ee} = European-European genotype, P_{en} = European-Native American genotype and P_{nn} = Native American-Native American genotype. Under the model, the fraction of the population that mates assortatively is denoted as α and the fraction that mates randomly is $1 - \alpha$. Taking the current generation ancestry genotype frequencies, the subsequent generation's ancestry genotype frequencies are calculated using the formulae:

$$P'_{aa} = (1-\alpha) \times a^2 + \alpha \times (P_{aa} + 0.25 \times P_{ae} + 0.25 \times P_{an})$$

$$P'_{ae} = (1-\alpha) \times 2ae + \alpha \times (0.5 \times P_{ae})$$

$$P'_{an} = (1-\alpha) \times 2an + \alpha \times (0.5 \times P_{an})$$

$$P'_{ee} = (1-\alpha) \times e^2 + \alpha \times (P_{ee} + 0.25 \times P_{ae} + 0.25 \times P_{en})$$

$$P'_{en} = (1-\alpha) \times 2en + \alpha \times (0.5 \times P_{en})$$

$$P'_{nn} = (1-\alpha) \times n^2 + \alpha \times (P_{nn} + 0.25 \times P_{an} + 0.25 \times P_{en})$$

Ancestry genotypes in each population were simulated for 20 generations, with the assumption of a generation time of 25 years, accounting for 500 years of elapsed time during the conquest and colonization of the Americas. The final ancestry genotype frequencies after the 20 generations were used to calculate the simulated ancestry homozygosity and heterozygosity values. For each Latin American simulated population, random gene sets, ranging in size from 2 to 20, were created by subsampling genes in the simulation. A meta-analysis AMI value and P-value for each gene set was calculated using the fixed-effects model of the Mantel-Haenszel method.

Control 4: Permutation test for ancestry-based assortative mating

Permutation of random gene sets was used to generate null distributions of gene set AMI values expected given the observed genome-wide levels of ancestry-based assortative mating. This permutation controls for the genome-wide levels of ancestry homozygosity based on overall levels of ancestry similarity between couples in admixed Latin American populations. For these permutations, 10,000 sets of genes, of the same sizes as the gene sets curated for the polygenic traits analyzed here, were permutated by randomly selecting genes without replacement from across the genome, and AMI values for the random gene sets were calculated. The randomly permuted AMI distributions were compared to the observed AMI values for the significant polygenic trait gene sets to evaluate the extent to which observed trait gene set AMI values deviate from AMI values seen for random gene sets.

2. Supplementary Results

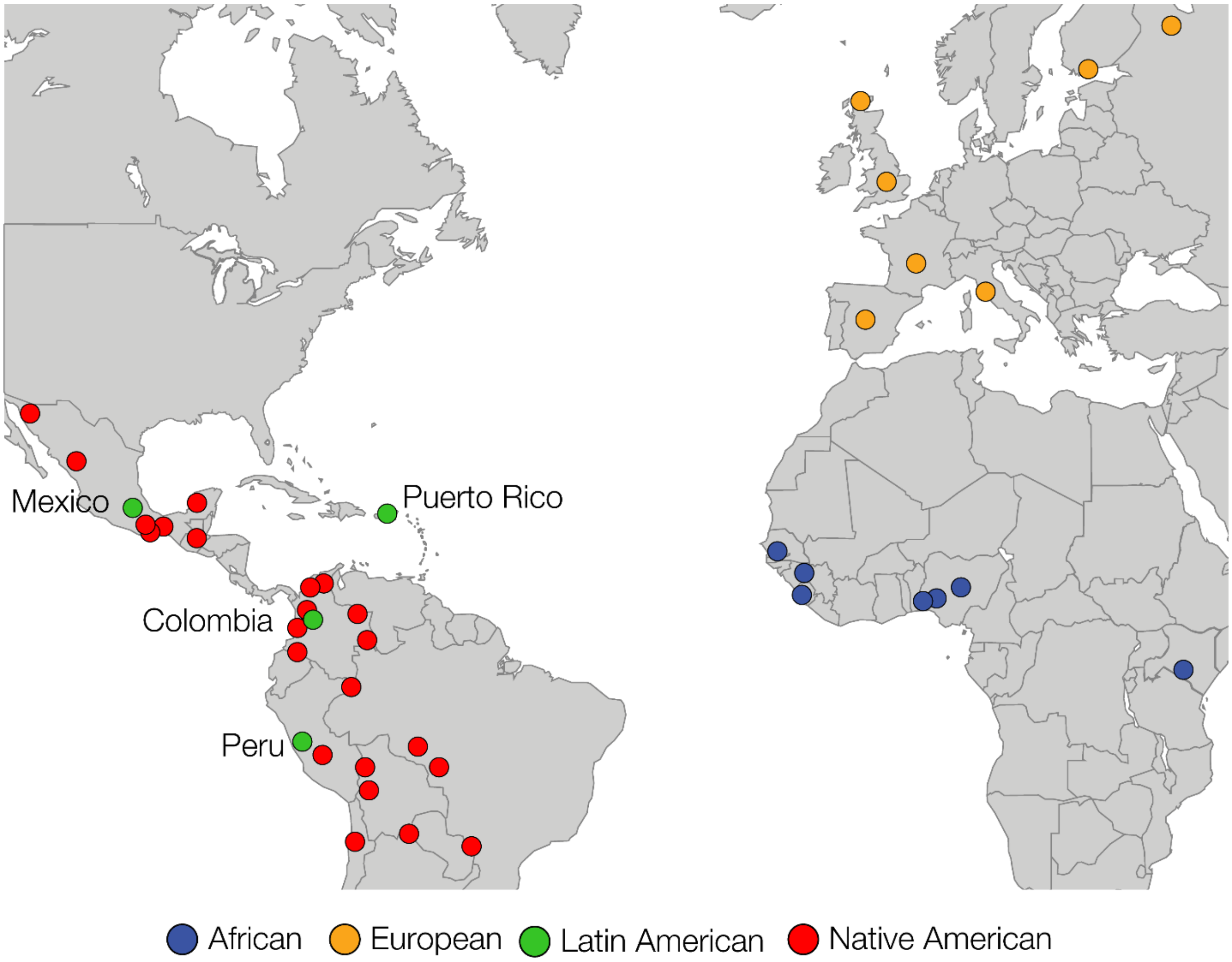

Figure S1. Global locations of the populations analyzed in this study. Global reference populations – African (blue), European (orange) and Native American (red) – were used to infer the continental ancestry proportions of the four admixed Latin American populations (green) studied here. Map adapted from https://commons.wikimedia.org/wiki/File:World_map_(Miller_cylindrical_projection,_blank).svg

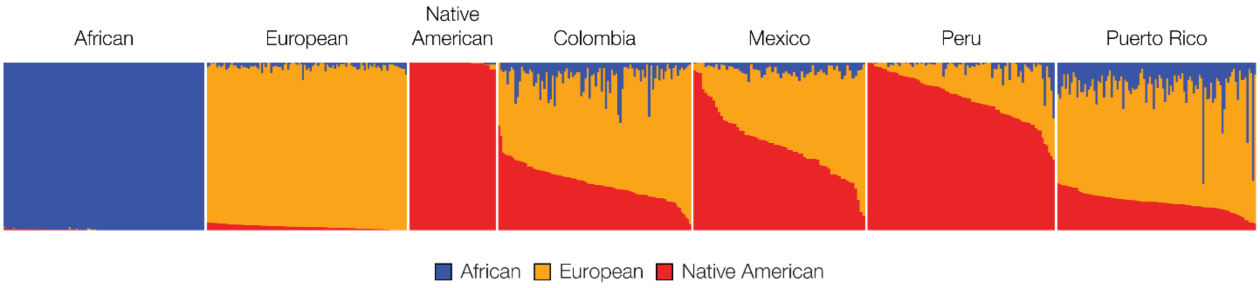

Figure S2. Three-way continental genetic ancestry for the four admixed Latin American populations analyzed in this study. ADMIXTURE plot showing genome-wide continental ancestry fractions for each individual in each of the four Latin American populations and for each individual in the global reference populations: African (blue), European (orange) and Native American (red).

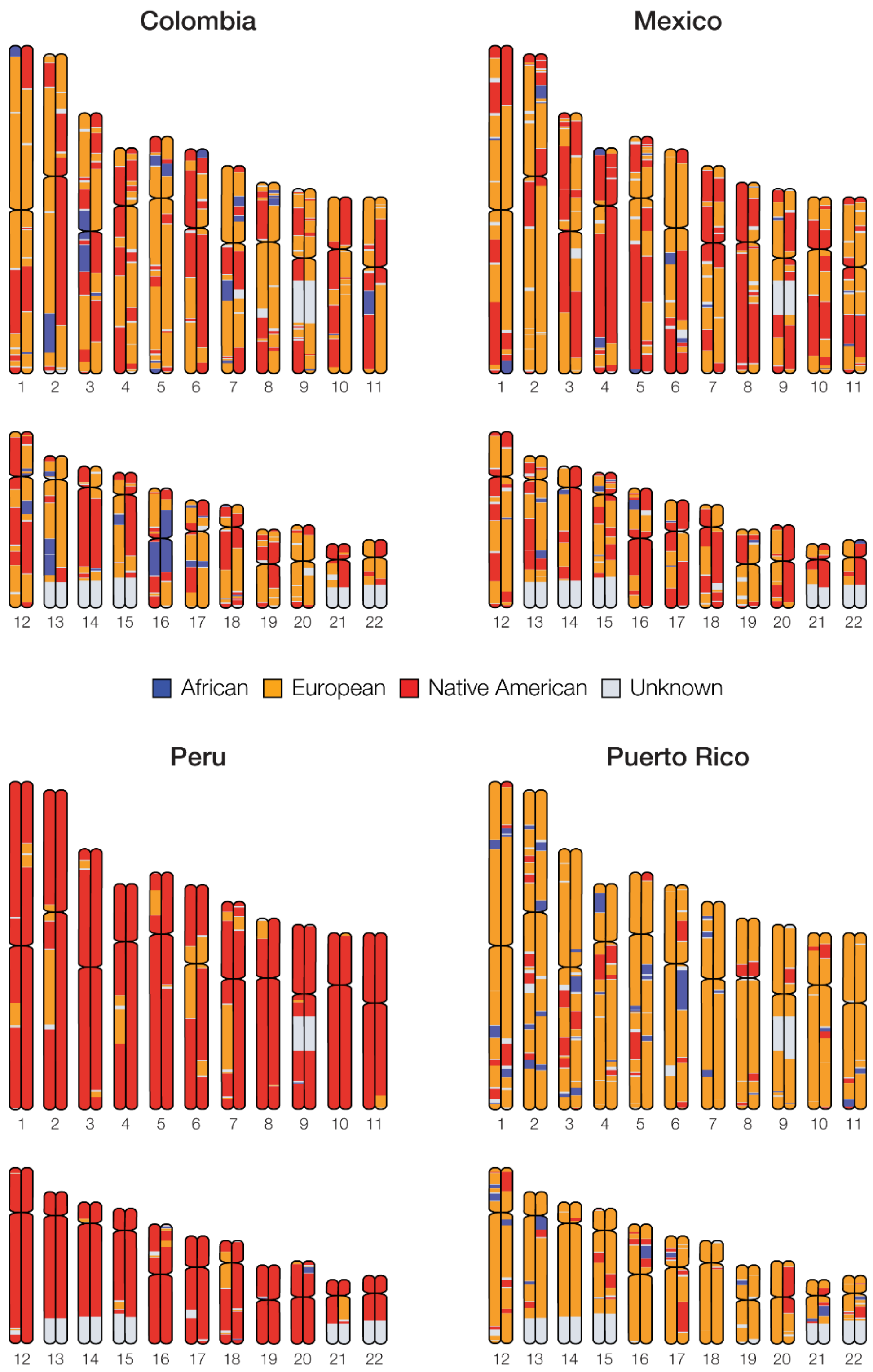

Figure S3. Local ancestry assignment with chromosome painting. Examples of local ancestry assignment chromosome paintings are shown for Colombia, Mexico, Peru and Puerto Rico. Examples correspond to genomes that have close to average ancestry proportions for each population.

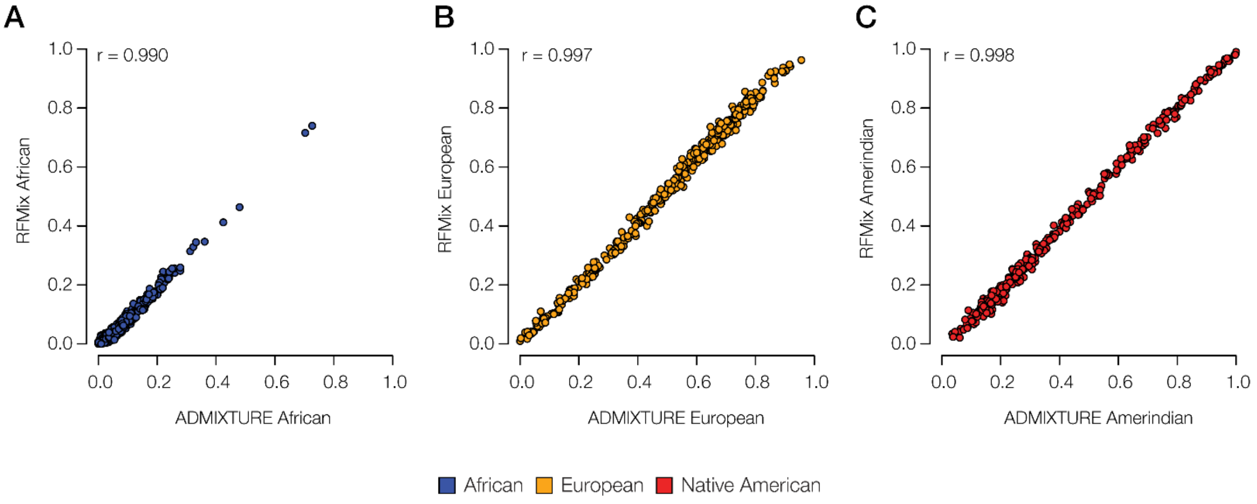

Figure S4. Comparison of ancestry fractions estimated by ADMIXTURE (global ancestry) versus RFMix (local ancestry). Genome-wide continental ancestry fractions were inferred for all individuals using the program ADMIXTURE, which estimates global ancestry fractions, and by summing the lengths of local ancestry-specific tracts (haplotypes) inferred from RFMix. Correlations between ADMIXTURE (x-axis) and RFMix (y-axis) ancestry estimates for individuals analyzed here are shown for (**A**) African (blue), (**B**) European (orange), and (**C**) Native American (red) ancestry. Pearson correlation *r*-values are shown for each ancestry.

Control 1: Evaluation of Hardy-Weinberg (HW)

We chose HW as an idealized population genetic model against which to test the observed distributions of local ancestry. Nevertheless, genome-wide patterns of local ancestry may deviate from HW depending on population history and demography. On the one hand, population structure may lead to the so-called Wahlund effect, yielding a genome-wide excess of homozygosity. On the other hand, very recent admixture could lead to a genome-wide excess of heterozygosity. We controlled for these two possibilities to validate the use of HW as a null model for the genome-wide distribution of local ancestry. Genome-wide patterns of homozygosity and heterozygosity were measured using the parameter Φ for the four admixed Latin American populations. Φ is expected to equal 2 under HW, whereas $\Phi<2$ indicates an excess of homozygosity and $\Phi>2$ indicates an excess of heterozygosity (Lachance, 2008). All four populations show genome-wide median values of Φ very close to 2 as well as genome-wide distributions centered around 2, in support of the use of HW as a null model for local ancestry distribution (Supplementary Figure S5A and S5B).

We also computed the genome-wide median values and distributions of heterozygosity to control for the possibility of recent admixture yielding extremely high values of ancestry heterozygosity genome-wide. Median values of heterozygosity are close to 0.5 for all four populations analyzed, and the genome-wide heterozygosity distributions do not show any evidence for extreme ancestry heterozygosity values caused by recent admixture (Supplementary Figure S5C and S5D). We further evaluated admixture timing for the four populations via analysis of the distribution of ancestry-specific haplotype lengths with the program TRACTS. The TRACTS analysis does not show any evidence for recent admixture among these populations; the inferred admixture events range from 8 to 15 generations ago, with an average of ~11 generations (Supplementary Figure S6). These estimates are consistent with previous studies as well as the known history of the region (Moreno-Estrada et al., 2013; Homburger et al., 2015; Conley et al., 2017). Taken together, the results of the heterozygosity and admixture timing analyses also support the use of HW as a null model for local ancestry distribution.

Control 2: Permutation of random mating

Results of the chromosome permutation control for random mating are shown as the expected AMI distributions in Figures 3A and 3B (see main body text for further description).

Control 3: Population genetic simulation of assortative mating

In addition to the permutation test, we also performed a simulation analysis using a population genetic model of assortative mating to assess the power of the AMI test statistic (Supplementary Figure S7). We were particularly interested in exploring the potential effects of different ancestry proportions among the populations analyzed here, and different gene set sizes, on computed AMI values. The population genetic simulation shows that our AMI test statistic is sensitive even when the fraction of the population that mates assortatively is low. We also found that AMI values are not biased in any particular direction based on the overall ancestry fractions observed for each population. For example, according to the AMI power simulation, Colombia should have the

highest overall AMI values, followed by Puerto Rico, Mexico, and Peru. This order is completely different from what is seen for the observed AMI values, where Mexico shows the highest mean value, followed by Peru, Colombia, and Puerto Rico (Figure 3B). The population genetic simulation does show that the size of the gene set being analyzed influences the sensitivity of the AMI test statistic. Larger gene sets show greater evidence for assortative mating at the same α parameter values compared with smaller gene sets.

Control 4: Permutation test for ancestry-based assortative mating

Results of the random genet set permutation control for assortative mating are shown as the permuted AMI distributions in Figure 4B (see main body text for further description).

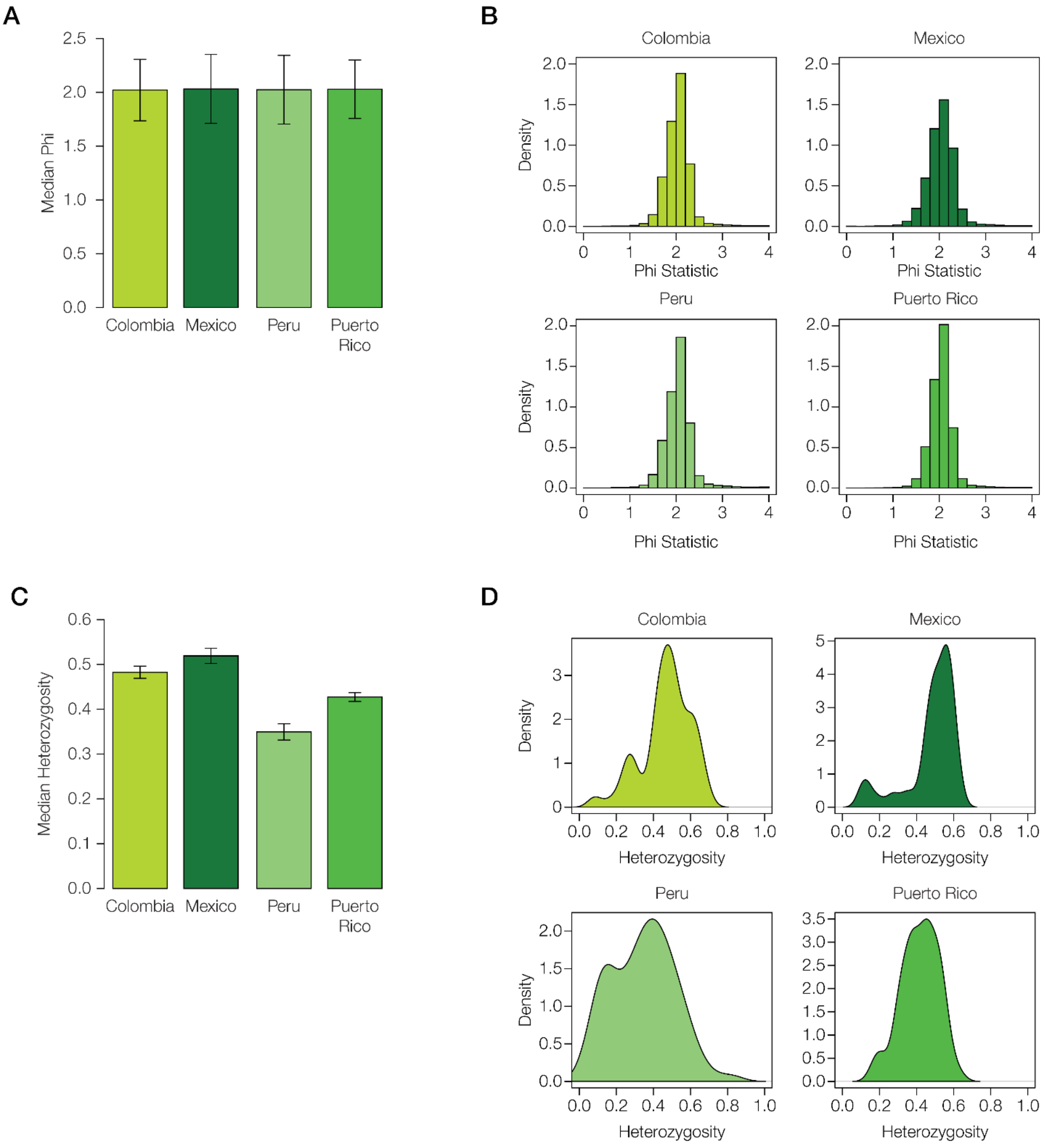

Figure S5. Genome-wide patterns of homozygosity and heterozygosity for the four admixed Latin American populations analyzed in this study. Genome-wide median values (**A**) and distributions (**B**) for the parameter Phi (Φ) were used to test for excess homozygosity and heterozygosity genome-wide. Genome-wide median values (**C**) and distributions (**D**) of heterozygosity were used to test for very recent admixture, which are expected to yield ~100% heterozygosity genome-wide.

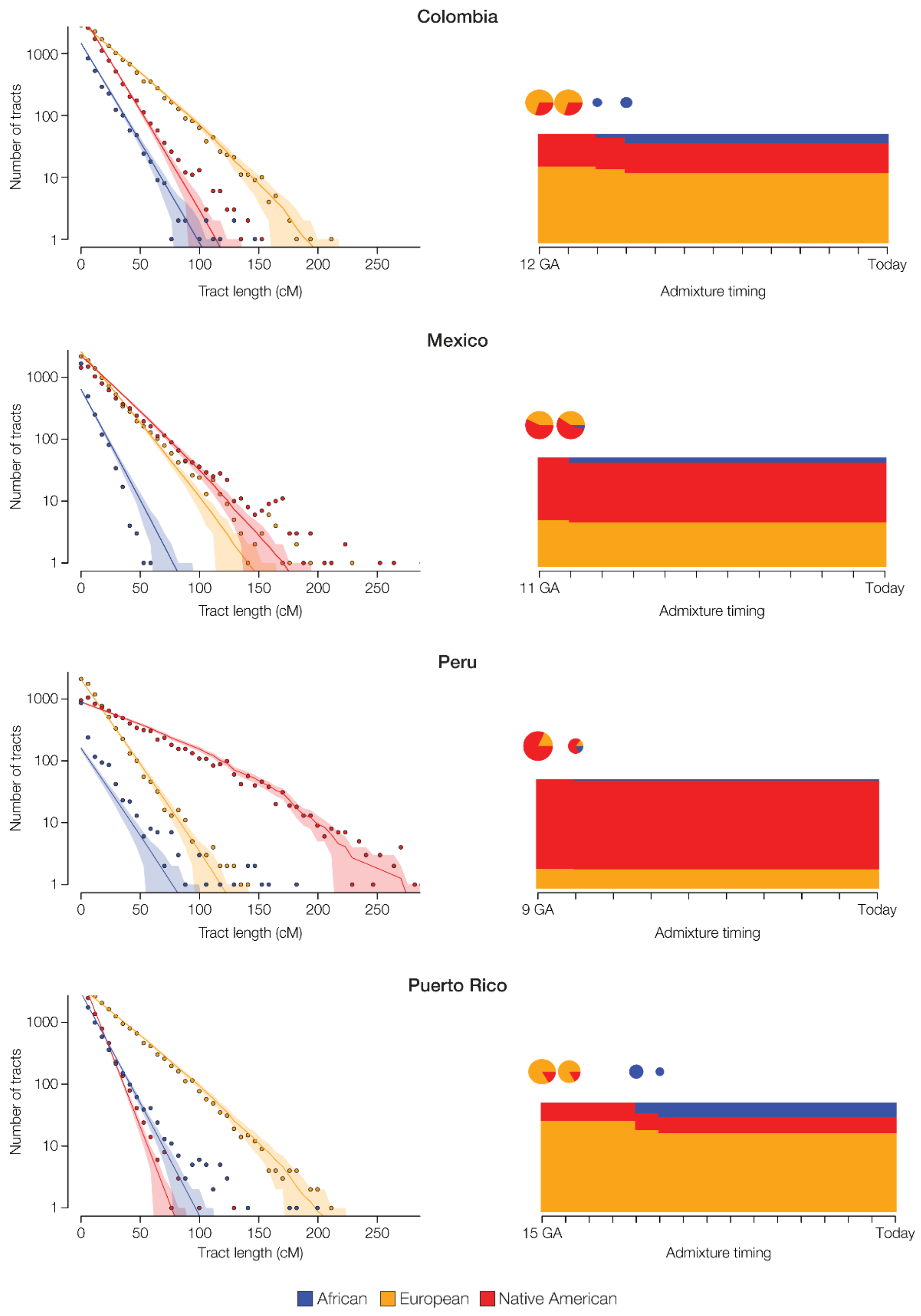

Figure S6. Admixture timing for the four admixed Latin American populations analyzed in this study. (Left panels) Observed (points) and predicted (solid lines) ancestry tract size distributions, with shaded areas representing 95% confidence intervals. (Right panels) Admixture event timings are shown together with ancestry proportions. Each inferred admixture event is indicated by a circle, which is scaled according to the size of the contribution to the population and also shows the relative ancestry proportions. The y-axes of the charts show the inferred continental ancestry fractions, and the x-axes show admixture timing as the number of generations ago (GA).

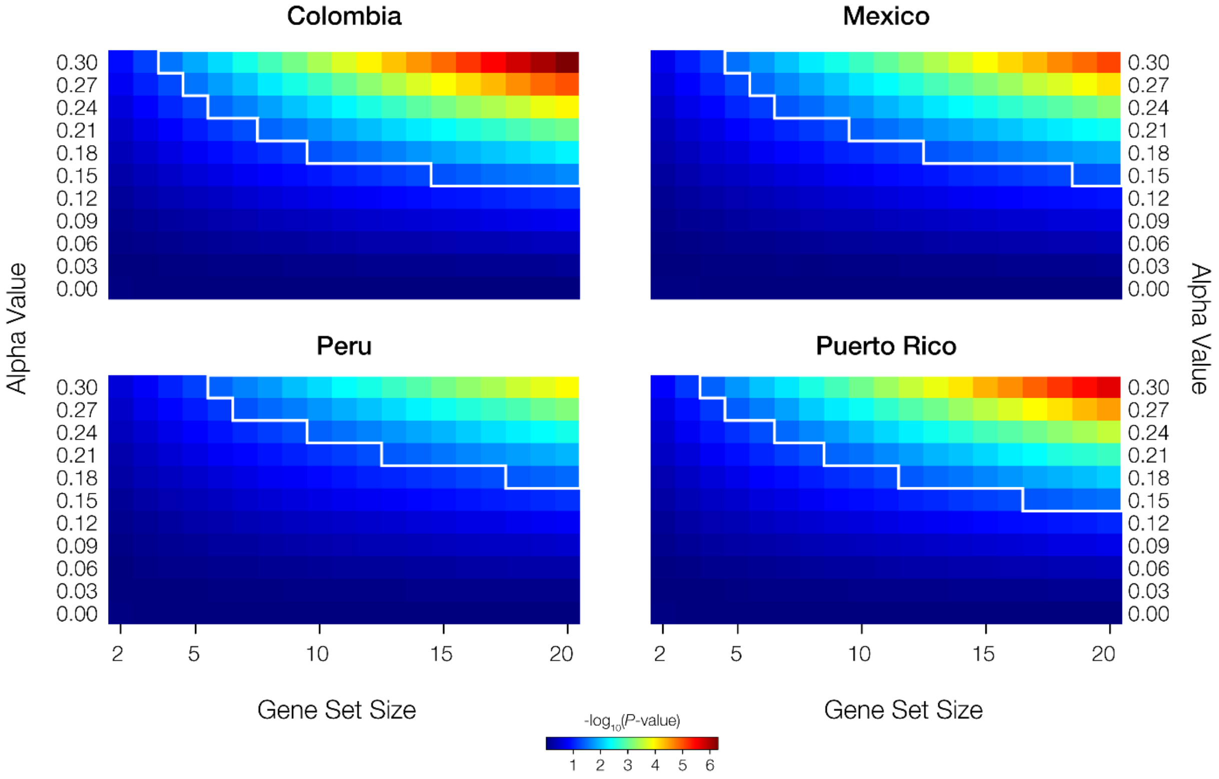

Figure S7. Simulation of the assortative mating index (AMI) test statistic under assortative mating. Assortative mating was modeled combining Hardy-Weinberg genotype expectations with a single parameter α that represents the fraction of the population that mates assortatively. Assortative mating α-values range from 0 (no assortative mating) to 0.3 (incomplete assortative mating) for polygenic phenotypes encoded by gene sets of n_g=2 to 20 genes. For each population, statistical significance P-values for AMI are plotted for all combinations of α and n_g, and the area of significant (P<0.05) AMI values is indicated.

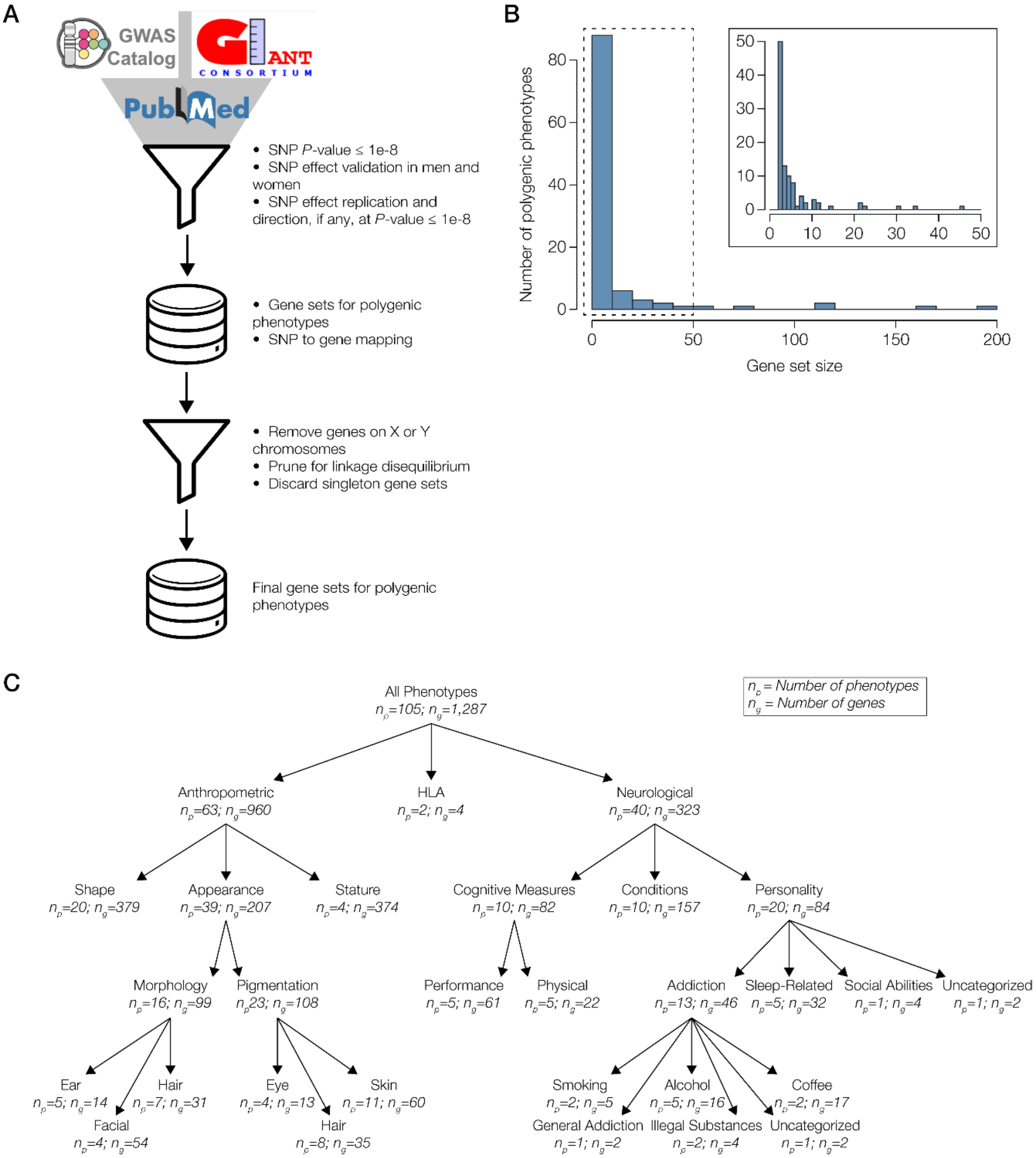

Figure S8. Polygenic phenotypes taken from genome-wide association studies (GWAS). (**A**) GWAS SNP-associations curated from (1) the NHGRI-EBI GWAS catalog, (2) the Genetic Investigation of ANthropometric Traits (GIANT) consortium, and (3) scientific literature indexed in PubMed were mapped to genes as described in the Methods in order to evaluate ancestry-based assortative mating on polygenic phenotypes. (**B**) Distribution of the number of genes per polygenic phenotype (trait). (**C**) Hierarchical organization scheme developed for the polygenic phenotypes analyzed here. The numbers of phenotypes (n_p) and total genes (n_g) are shown for each node in the scheme tree.

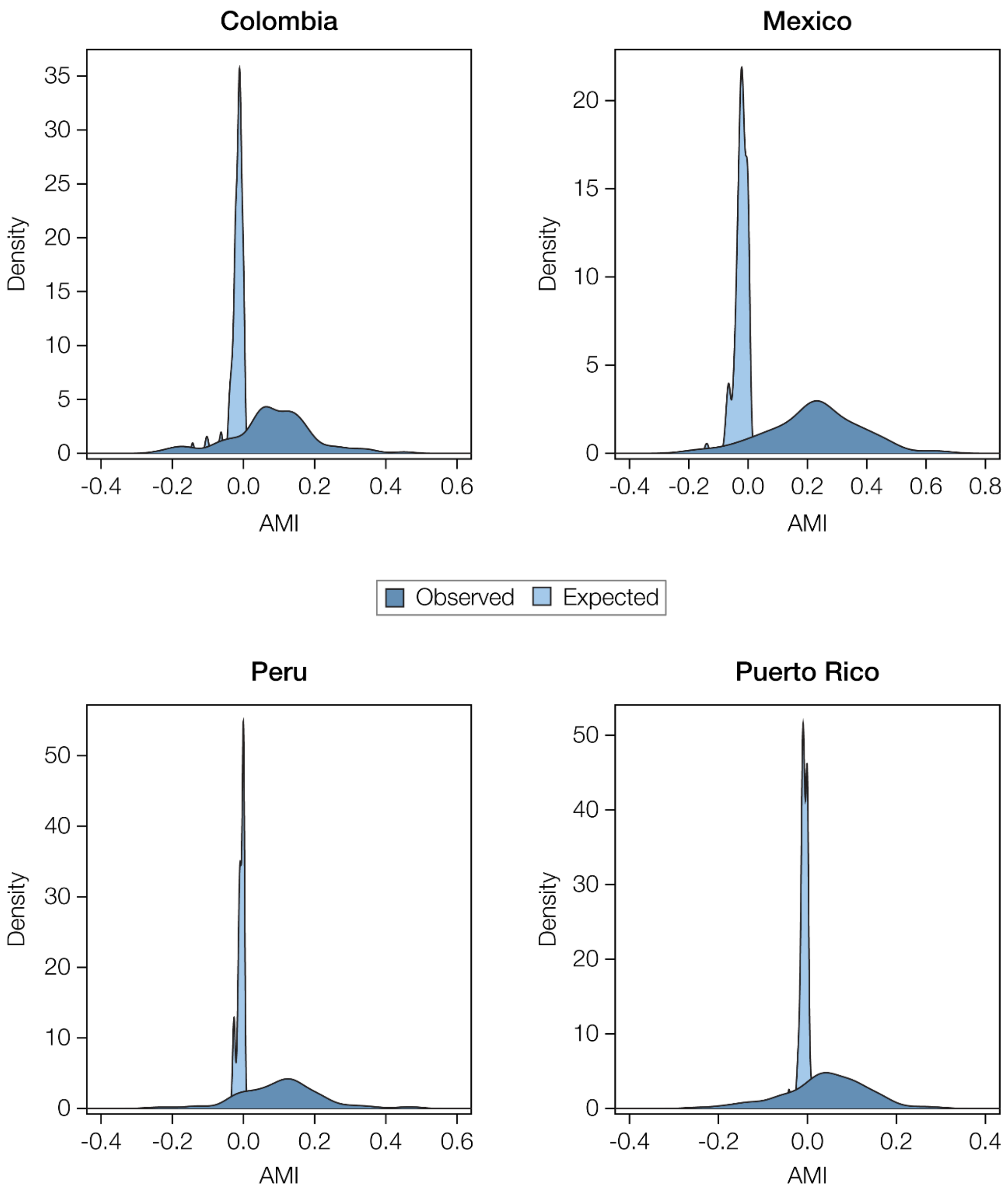

Figure S9. Distributions of observed (dark blue) versus expected (light blue) AMI values for the four admixed Latin American populations analyzed here.

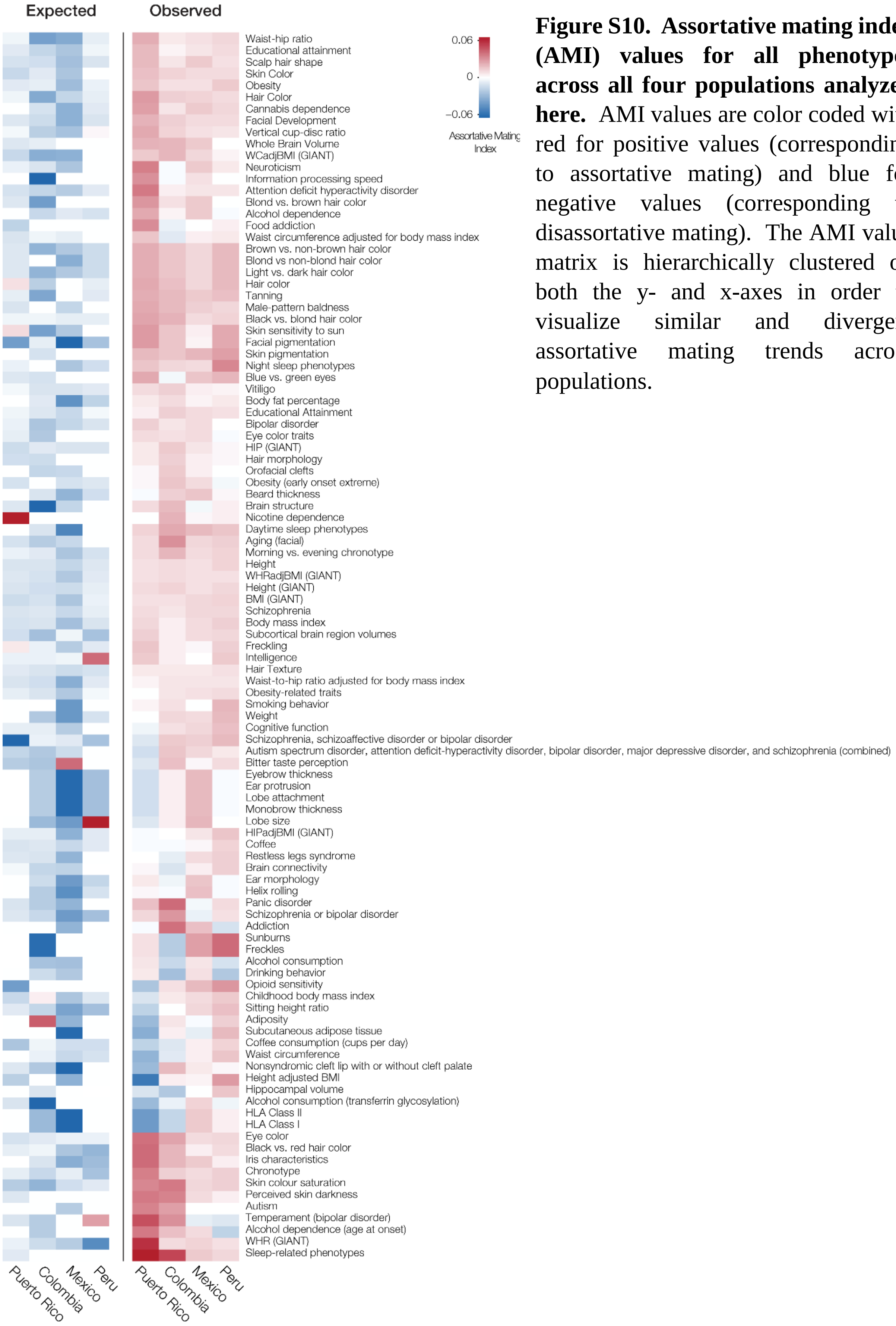

Figure S10. Assortative mating index (AMI) values for all phenotypes across all four populations analyzed here. AMI values are color coded with red for positive values (corresponding to assortative mating) and blue for negative values (corresponding to disassortative mating). The AMI value matrix is hierarchically clustered on both the y- and x-axes in order to visualize similar and divergent assortative mating trends across populations.

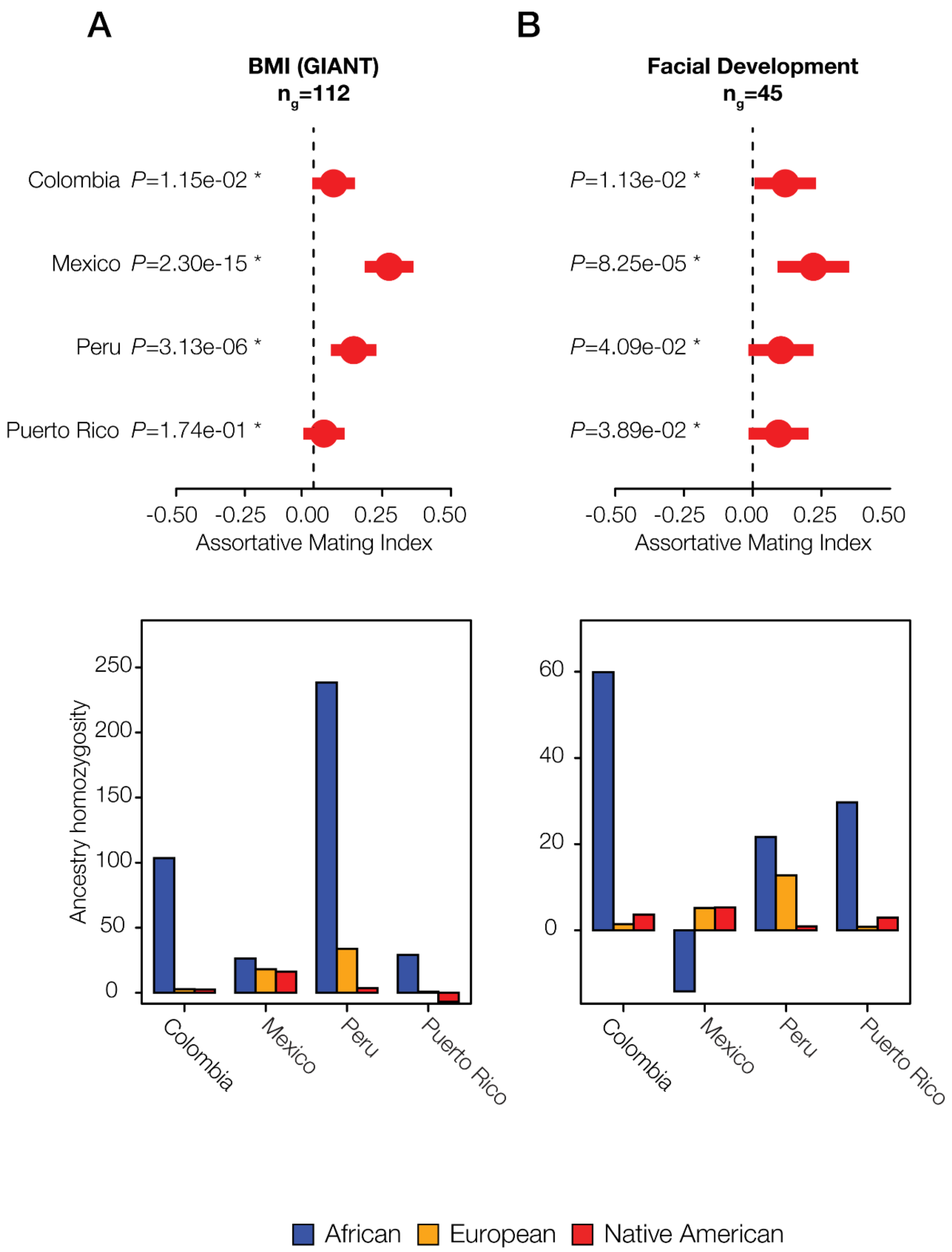

Figure S11. Individual examples of ancestry-based assortative mating. Results of meta-analysis of (dis)assortative mating of polygenic phenotypes along with their ancestry drivers are shown for (**A**) body mass index, and (**B**) facial development. The meta-analysis plots show pooled AMI odds ratio values along with their 95% CIs and *P*-values. Stars indicate false discovery rate *q*-values<0.05. The ancestry driver plots show the extent to which individual ancestry components have an excess or deficit of homozygosity.

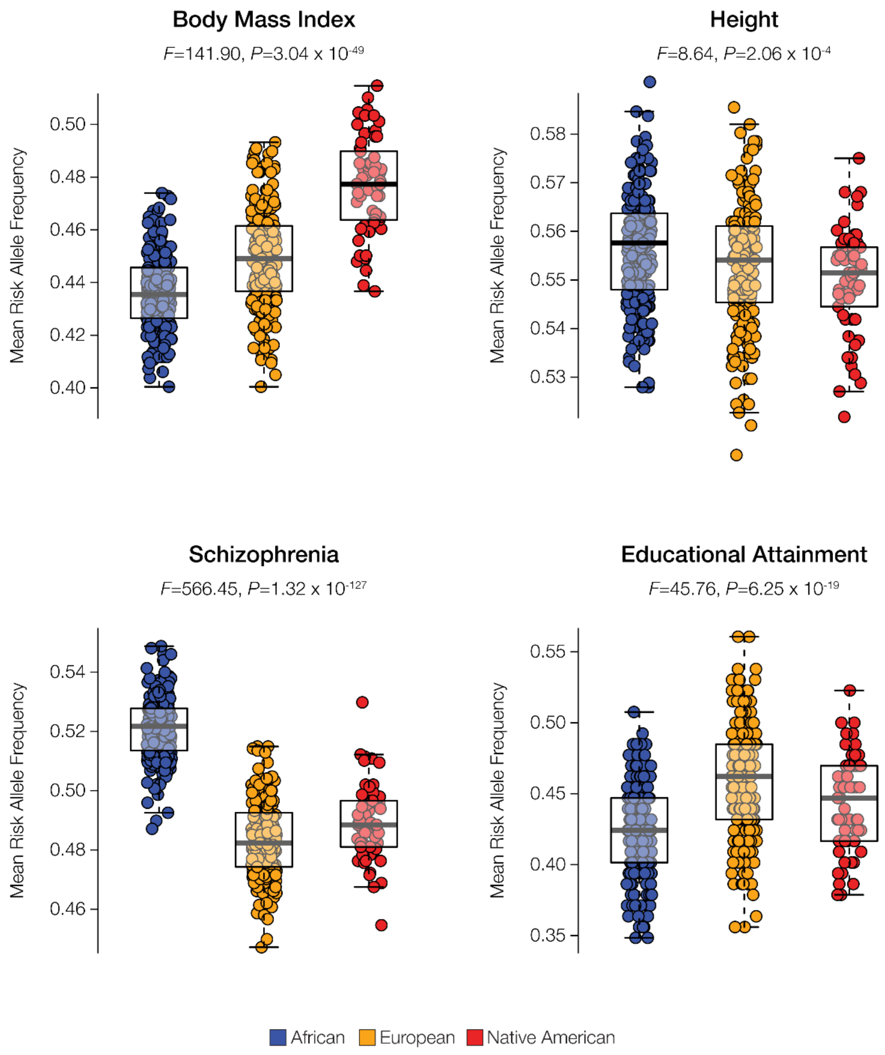

Figure S12. Genetic variation in trait-specific SNP frequencies across continental ancestry groups. Average risk allele frequency distributions are shown for the top four ancestry-based assortative mating traits (see Figure 4A). Average SNP risk allele frequencies for each trait were computed using the 1000 Genomes Project data for individuals from African (GWD and YRI), European (CEU and IBS), and Native American (MXL and PEL) populations. To approximate Native American ancestry, only Mexican (MXL) and Peruvian (PEL) individuals with >75% Native American ancestry were chosen (see Figure S2). ANOVA was used to evaluate the significance of the differences in the ancestry-specific distributions for each trait (see *F*- and *P*-values for each plot).

Table S1. References and values for phenotypes with significant AMI values and population variance.

Phenotypes with significant AMI values						
**Phenotype**	**Population**	**Database[1]**	**PMID(s)[2]**	**AMI value[3]**	***q*-value[4]**	**Wilcoxon *P*-value[5]**
BMI	Mexico	GIANT	20935630, 22982992, 23563607, 23754948, 25673413	0.27	2.42E-13	0
Schizophrenia	Mexico	GWAS Catalog	19571808, 19571811, 21682944, 21926974, 22037552, 22037555, 22688191, 22883433, 23142968, 23894747, 23974872, 24043878, 25056061, 26198764	0.26	6.99E-12	0
Body mass index	Mexico	GWAS Catalog	17434869, 18454148, 19079260, 19079261, 20935630, 22344219, 22344221, 22982992, 23563607, 23583978, 23669352, 24064335, 24348519, 24861553, 25673413, 25953783	0.24	4.08E-07	0
Height	Peru	GWAS Catalog	17767157, 18193045, 18391950, 18391951, 18391952, 18952825, 19343178, 19396169, 19570815, 19729412, 19893584, 20189936, 20397748, 20881960, 21998595, 22021425, 23456168, 23563607, 25282103, 25429064	0.13	3.62E-06	0
Height	Peru	GIANT	20881960, 22982992, 23563607, 23754948, 25282103	0.12	7.27E-05	0
BMI	Peru	GIANT	20935630, 22982992, 23563607, 23754948, 25673413	0.15	1.10E-04	0
Facial Development	Mexico	Custom	24651127	0.22	1.44E-03	1.46E-02
Height	Colombia	GIANT	20881960, 22982992, 23563607, 23754948, 25282103	0.11	1.60E-03	7.53E-243
Body mass index	Peru	GWAS Catalog	17434869, 18454148, 19079260, 19079261, 20935630, 22344219,	0.15	2.11E-03	0

			22344221, 22982992, 23563607, 23583978, 23669352, 24064335, 24348519, 24861553, 25673413, 25953783			
Schizophrenia	Peru	GWAS Catalog	19571808, 19571811, 21682944, 21926974, 22037552, 22037555, 22688191, 22883433, 23142968, 23894747, 23974872, 24043878, 25056061, 26198764	0.12	3.10E-03	7.28E-239
Educational Attainment	Mexico	Custom	27225129	0.22	4.32E-03	1.82E-10
Morning vs. evening chronotype	Mexico	GWAS Catalog	26955885, 26835600	0.22	1.92E-01	4.93E-29
Combined neurological disorders[6]	Mexico	GWAS Catalog	23453885	0.26	2.18E-01	0
Beard thickness	Mexico	GWAS Catalog	26926045	0.39	2.18E-01	0
Bipolar disorder	Mexico	GWAS Catalog	17554300, 17486107, 18711365, 19416921, 21926972, 21353194, 22205951, 22182935, 21254220, 24618891	0.23	2.18E-01	0
Childhood body mass index	Mexico	GWAS Catalog	26604143	0.24	2.18E-01	9.93E-20
Helix rolling	Mexico	GWAS Catalog	26105758	0.41	2.18E-01	8.17E-43
Lobe size	Mexico	GWAS Catalog	26105758	0.48	2.18E-01	0
Scalp hair shape	Mexico	GWAS Catalog	26926045	0.35	2.20E-01	0
Chronotype	Mexico	GWAS Catalog	26955885, 26835600	0.25	2.60E-01	0

Phenotypes with significant AMI population variance

**Phenotype**	**Database[1]**	**PMID(s)[2]**	**AMI variance[7]**	***q*-value[8]**
Freckles	GWAS Catalog	23548203	1.41E-01	0

Sunburns	GWAS Catalog	17952075, 18488028	1.41E-01	0
Addiction	GWAS Catalog	23533358	7.23E-02	1.10E-103
HLA Class I	Custom	20356336	6.25E-02	2.20E-68
HLA Class II	Custom	20356336	6.25E-02	2.20E-68
Opioid sensitivity	GWAS Catalog	24143882	6.20E-02	6.53E-67
Lobe size	GWAS Catalog	26105758	5.85E-02	3.23E-56
Ear protrusion	GWAS Catalog	26105758	5.72E-02	1.67E-52
Eyebrow thickness	GWAS Catalog	26926045	5.72E-02	1.67E-52
Lobe attachment	GWAS Catalog	26105758	5.72E-02	1.67E-52
Monobrow thickness	GWAS Catalog	26926045	5.72E-02	1.67E-52
Height adjusted BMI	GWAS Catalog	25044758	5.36E-02	4.79E-43
Temperament (bipolar disorder)	GWAS Catalog	22365631	4.81E-02	6.37E-30
Drinking behavior	GWAS Catalog	21372407, 23364009	4.75E-02	8.38E-29
Helix rolling	GWAS Catalog	26105758	4.44E-02	1.04E-22
Ear morphology	GWAS Catalog	26105758	4.10E-02	6.61E-17
Body fat percentage	GWAS Catalog	26833246	8.83E-04	2.22E-16
Black vs. red hair color	GWAS Catalog	18483556	1.63E-03	2.96E-15

Vitiligo	GWAS Catalog	21326295, 22561518, 19890347, 20410501, 20526339, 22951725	1.94E-03	8.26E-15
Eye color	GWAS Catalog	20585627, 23118974, 23548203	2.27E-03	2.42E-14

[1] Database source for the trait SNP-associations and gene sets. 'Custom' refers to SNP-associations mined from the literature.

[2] PubMed identifiers for the publications where the trait SNP-associations are reported.

[3] Value of the assortative mating index (AMI) test statistic for the phenotype gene set.

[4] False discovery rate *q*-value for the significance of the AMI value.

[5] *P*-value of the Wilcoxon rank sum test comparing the permuted AMI statistics to the observed AMI statistic

[6] Combined neurological disorders: Autism spectrum disorder, attention deficit-hyperactivity disorder, bipolar disorder, major depressive disorder, and schizophrenia (combined)

[7] Variance of the AMI test statistics for the phenotype gene set across all 4 Latin American populations.

[8] False discovery rate *q*-value for the variance of the AMI test statistics across the 4 Latin American populations.

Table S2. Ancestry differences for phenotypes implicated in assortative mating (i.e. mate choice) in admixed Latin American populations.

Trait	Ancestry[1] African	European	Native American	Variance	Source
**Height (Male)**<br>**Height (Female)**	167 cm<br>160 cm	175 cm<br>162 cm	164 cm<br>151 cm	34 cm<br>38 cm	https://en.wikipedia.org/wiki/List_of_average_human_height_worldwide
**Body Mass Index[2] (Male)**<br>**Body Mass Index[2] (Female)**	22.8<br>24.0	27.4<br>26.0	25.7<br>26.9	5.4<br>2.2	http://www.who.int/nmh/publications/ncd-status-report-2014/en/
**Schizophrenia[3]**	247	186	253	1374	http://www.who.int/healthinfo/global_burden_disease/2004_report_update/en/
**Educational Attainment[4]**	44%	67%	74%	246.3	https://www.education-inequalities.org/indicators/comp_upsec_v2

[1] Ancestry-specific measures are based on values from Nigeria (African), Spain (European), and Peru (Native American). Nigeria and Spain are chosen, as they are known to provide the highest African and European ancestry components to admixed Latin American countries. Peru is chosen as it is known to have the highest Native American ancestry proportion in Latin America (ref http://journals.plos.org/plosgenetics/article?id=10.1371/journal.pgen.1005602).

[2] Body mass index is measured as a person's mass (weight) divided by the square of their height.

[3] Schizophrenia is measured as the age-standardized disability-adjusted life years (DALY) rates per 100,000 inhabitants (recorded in 2004).

[4] Educational attainment is the percentage of people aged 3-5 years above upper secondary school graduation age who have completed upper secondary school.

3. References

Conley, A.B., Rishishwar, L., Norris, E.T., Valderrama-Aguirre, A., Marino-Ramirez, L., Medina-Rivas, M.A., et al. (2017). A Comparative Analysis of Genetic Ancestry and Admixture in the Colombian Populations of Choco and Medellin. *G3 (Bethesda)* 7(10)**,** 3435-3447. doi: 10.1534/g3.117.1118.

Homburger, J.R., Moreno-Estrada, A., Gignoux, C.R., Nelson, D., Sanchez, E., Ortiz-Tello, P., et al. (2015). Genomic Insights into the Ancestry and Demographic History of South America. *PLoS Genet* 11(12)**,** e1005602. doi: 10.1371/journal.pgen.1005602.

Lachance, J. (2008). A fundamental relationship between genotype frequencies and fitnesses. *Genetics* 180(2)**,** 1087-1093. doi: 10.1534/genetics.108.093518.

Moreno-Estrada, A., Gravel, S., Zakharia, F., McCauley, J.L., Byrnes, J.K., Gignoux, C.R., et al. (2013). Reconstructing the population genetic history of the Caribbean. *PLoS Genet* 9(11)**,** e1003925. doi: 10.1371/journal.pgen.1003925.
